# A Genome-Wide Analysis of Adhesion in *Caulobacter crescentus* Identifies New Regulatory and Biosynthetic Components for Holdfast Assembly

**DOI:** 10.1128/mBio.02273-18

**Published:** 2019-02-12

**Authors:** David M. Hershey, Aretha Fiebig, Sean Crosson

**Affiliations:** a Department of Biochemistry and Molecular Biology, University of Chicago, Chicago, Illinois, USA; b Department of Microbiology, University of Chicago, Chicago, Illinois, USA; Indiana University Bloomington; Massachusetts Institute of Technology

**Keywords:** BarSeq, *Caulobacter*, adhesion, holdfast, pilus, polysaccharide

## Abstract

Due to their intimate physical interactions with the environment, surface polysaccharides are critical determinants of fitness for bacteria. Caulobacter crescentus produces a specialized structure at one of its cell poles called the holdfast that enables attachment to surfaces. Previous studies have shown that the holdfast is composed of carbohydrate-based material and identified a number of genes required for holdfast development. However, incomplete information about its chemical structure, biosynthetic genes, and regulatory principles has limited progress in understanding the mechanism of holdfast synthesis. We leveraged the adhesive properties of the holdfast to perform a saturating screen for genes affecting attachment to cheesecloth over a multiday time course. Using similarities in the temporal profiles of mutants in a transposon library, we defined discrete clusters of genes with related effects on cheesecloth colonization. Holdfast synthesis, flagellar motility, type IV pilus assembly, and smooth lipopolysaccharide (SLPS) production represented key classes of adhesion determinants. Examining these clusters in detail allowed us to predict and experimentally define the functions of multiple uncharacterized genes in both the holdfast and SLPS pathways. In addition, we showed that the pilus and the flagellum control holdfast synthesis separately by modulating the holdfast inhibitor *hfiA.* This report defines a set of genes contributing to adhesion that includes newly discovered genes required for holdfast biosynthesis and attachment. Our data provide evidence that the holdfast contains a complex polysaccharide with at least four monosaccharides in the repeating unit and underscore the central role of cell polarity in mediating attachment of C. crescentus to surfaces.

## Abstract (precis)

Bacteria routinely encounter biotic and abiotic materials in their surrounding environments, and they often enlist specific behavioral programs to colonize these materials. Adhesion is an early step in colonizing a surface. Caulobacter crescentus produces a structure called the holdfast which allows this organism to attach to and colonize surfaces. To understand how the holdfast is produced, we performed a genome-wide search for genes that contribute to adhesion by selecting for mutants that could not attach to cheesecloth. We discovered complex interactions between genes that mediate surface contact and genes that contribute to holdfast development. Our genetic selection identified what likely represents a comprehensive set of genes required to generate a holdfast, laying the groundwork for a detailed characterization of the enzymes that build this specialized adhesin.

## INTRODUCTION

The bacterial cell envelope is a highly dynamic structure that is essential for growth and division (1). Carbohydrate-based compounds often form the outermost layer of the envelope, comprising a specialized surface that each cell displays to the surrounding environment (2). The roles of surface polysaccharides such as capsules, exopolysaccharides, and O-antigens in promoting colonization of preferred niches are well established for both free-living and host-associated bacteria (3–5). However, the enzymes that synthesize and export these polysaccharides have been difficult to characterize due to the chemical complexity of the metabolic intermediates (6). Defining the molecular details of how extracellular carbohydrates are produced is critical to understanding bacterial colonization and how it can be controlled.

**FIG 1:**
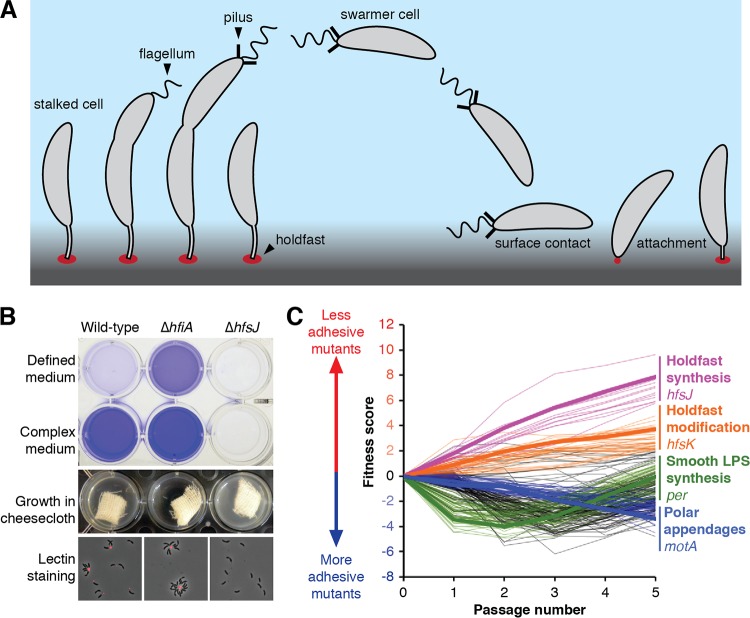
A genome-wide screen for holdfast biosynthesis genes identified multiple classes of mutants affecting adhesion. (A) During the dimorphic C. crescentus life cycle, each cell division produces a motile swarmer cell and a sessile stalked cell. Swarmer cells stop swimming, shed their flagellum and pili, and develop into stalked cells before dividing. Stalked cells can adhere strongly to exogenous surfaces using a specialized material called the holdfast. (B) The holdfast can be visualized by staining with fluorescently labeled wheat germ agglutinin (fWGA), and biofilm formation can be quantified by crystal violet staining of attached cells. The Δ*hfiA* cells overproduced holdfast and were hyperadhesive. The Δ*hfsJ* cells did not produce holdfasts and were nonadhesive. Attachment was reduced in defined medium due to high levels of *hfiA* expression. (C) Fitness profiles for the 250 genes with the strongest adhesion phenotypes in the cheesecloth passaging experiment. Lines are drawn to connect mean fitness scores at days 0 through 5 for each of the 250 genes. Genes in the four major fitness clusters are colored as indicated in the legend, with a specific example listed. Genes shown in black do not fit into any of the four clusters.

The aquatic bacterium Caulobacter crescentus has a dimorphic lifestyle characterized by an association with exogenous surfaces. Division in C. crescentus is asymmetric and produces two distinct cell types: a chemotactic swarmer cell and a sessile, replication-competent stalked cell (7). In response to environmental and developmental signals, the swarmer cell sheds its flagellum, disassembles its pili, and transitions into its stalked-cell form before dividing (8). Stalked cells are named for a specialized envelope extension called the stalk that emerges from the old pole after disassembly of the flagellum and pili. During the swarmer-cell-to-stalked-cell transition, cells often produce a polysaccharide-rich matrix called the holdfast at the site of stalk development (9). This highly adhesive material allows C. crescentus to form essentially permanent interactions with exogenous surfaces (Fig. 1A) (10).

Due to the irreversible nature of surface attachment in C. crescentus, the timing of holdfast production is tightly controlled. Only small proportions of cells produce a holdfast under conditions of growth in defined medium, whereas nearly all cells produce a holdfast under conditions of growth in complex medium (11). This effect is due to elevated expression of the *holdfast inhibitor A* (*hfiA*) gene in defined medium (Fig. 1B). *hfiA* expression is also coordinated with the cell cycle. Its transcript levels drop during the swarmer to stalked transition, which corresponds to the developmental stage at which holdfasts begin to appear. Numerous signaling pathways target the *hfiA* promoter, allowing the cell to integrate environmental, nutritional, and developmental cues into a single output that regulates adhesion (11, 12). In yet another regulatory regime, holdfast synthesis can be induced when a swarmer cell encounters a surface (13). Physical disruption of flagellar rotation or pilus retraction upon surface contact stimulates the production of a holdfast (14, 15). How the numerous regulatory pathways converge to control holdfast development remains unclear, but the complexity of these networks reflects the significance of committing to a surface-associated lifestyle.

Genetic analysis of nonadhesive mutants indicates that the holdfast is a polysaccharide-based material. The holdfast synthesis (*hfs*) genes include those encoding predicted glycosyltransferases, carbohydrate modification factors, and components of a *wzy-*type polysaccharide assembly pathway (16–19). *wzy*-dependent carbohydrate assembly utilizes a lipid carrier known as undecaprenylpyrophosphate (UPP) on which glycosyltransferases assemble an oligosaccharide repeating unit in the cytoplasm (20). The resulting glycolipid is flipped from the cytoplasmic face of the inner membrane to the periplasmic face where the oligosaccharide is polymerized and exported to the cell surface (21). The *wzy* mechanism is used to produce an impressive diversity of polysaccharides and is broadly conserved among bacteria (22). Thus, characterization of enzymes involved in the biosynthesis of the holdfast has the potential to uncover broadly applicable principles about how bacteria produce carbohydrate polymers.

The chemical nature of the holdfast matrix remains poorly characterized. The holdfast binds to *N*-acetylglucosamine (GlcNAc)-specific lectin wheat germ agglutinin (WGA) and is sensitive to the GlcNAc-specific hydrolases chitinase and lysozyme, indicating that GlcNAc is a component of the matrix (23). Little other information about the carbohydrate content has been reported. Extracellular DNA and an unidentified protein component(s) contribute to the stiffness of the holdfast, but only mutations in polysaccharide biosynthesis genes or genes encoding pleiotropic regulators of cell polarity abolish holdfast production (24). Currently, three glycosyltransferase steps are known to be required for holdfast synthesis. An initial reaction carried out by a genetically redundant HfsE, PssY, or PssZ enzyme is thought to be followed by the activities of HfsG and HfsJ, suggesting that the polysaccharide may be composed of a trisaccharide repeat (11, 18). However, new *hfs* genes continue to be discovered, hinting that additional glycosyltransferases may remain unidentified (11, 25). Uncertainty about both the composition of the holdfast and the saturation of screens for *hfs* genes presents a major obstacle to characterizing enzymatic reactions in the pathway.

Here, we utilized saturating transposon mutagenesis to probe holdfast production at the genome scale. We developed a barcoded transposon library in C. crescentus and enriched for nonadhesive mutants by performing passaging across multiple days in the presence of cheesecloth. We discovered a surprising number of genes with distinct adhesion phenotypes that ranged from hyperadhesive to nonadhesive. We found that disrupting the smooth lipopolysaccharide (SLPS) leads to a holdfast-independent form of ectopic adhesion that is not restricted to the cell pole but is instead mediated throughout the cell surface. The temporal adhesion profiles of known SLPS mutants were used to identify and characterize new genes in the SLPS pathway. The same fitness correlation approach was used to place previously uncharacterized genes in the holdfast pathway. We further demonstrated that disrupting the assembly of polar surface appendages modulates the activity of the *hfiA* holdfast inhibitor. In particular, individual mutations in the pilus machinery had a range of adhesion phenotypes, suggesting that distinct intermediates in the pilus assembly pathway have opposing effects on *hfiA*. On the basis of our comprehensive analysis of holdfast regulation, biosynthesis, and assembly, we propose a model that outlines the sequence of enzymatic steps required to produce the holdfast polysaccharide.

## RESULTS

### A screen for mutants with altered adhesion characteristics.

The holdfast promotes adhesion of C. crescentus cells to a variety of surfaces (26). We reasoned that adhesive cells could be depleted from liquid cultures by adding an attachment substrate with a sufficiently large surface area. Cheesecloth has been used in this manner to enrich for holdfast mutants in both C. crescentus and Asticcacaulis biprosthecum, another stalked bacterium in the *Caulobacteraceae* clade (27, 28). Adding sterile cheesecloth to wild-type C. crescentus cultures decreased the turbidity of the medium by titrating adhesive cells from the broth. This effect was amplified in the hyperadhesive Δ*hfiA* strain but not observed in the holdfast-deficient Δ*hfsJ* strain, demonstrating the effectiveness of cheesecloth at capturing cells with a holdfast (Fig. 1B). We concluded that growth in the presence of cheesecloth could be used as the basis of a selection to identify mutants defective in adhesion.

Saturating transposon mutagenesis coupled with transposon insertion sequencing (TnSeq) offers the advantage of scoring phenotypes for all nonessential genes in the genome simultaneously (29). Thus, combining TnSeq-based mutant profiling with cheesecloth depletion seemed appropriate to perform a saturating screen for holdfast biosynthesis genes and to identify missing biosynthesis factors. We developed a randomly barcoded transposon library in C. crescentus to enable the use of BarSeq (30) for profiling mutant fitness. Adhesive cells were depleted by passaging the library in cheesecloth for five cycles. During each passage, the library was cultured for 24 h in the presence of cheesecloth, after which the unattached cells in the medium were used to reinoculate a fresh culture containing cheesecloth. An aliquot of unattached cells in the medium was also harvested for BarSeq analysis. Three passaging experiments with cheesecloth were performed in parallel. To discriminate mutants with adhesion defects from those with growth defects, we also performed three passaging experiments without cheesecloth for comparison (see Table S1 in the supplemental material).

10.1128/mBio.02273-18.5 TABLE S1 Samples used for BarSeq analysis of gene fitness across cheesecloth passages. The “Index” column presents the TruSeq indices used to demultiplex the samples after sequencing. “Cheese” and “PYE” represent passages with and without cheesecloth, respectively. The numbers indicate which of the five passages each sample represents, and the letters indicate which of the three replicates is represented. The data presented in the “Proportion of reads in top percentile” column were calculated by ranking the barcodes in each sample by their abundance and determining the proportion of reads that mapped to the top 1% of barcodes. Download Table S1, DOCX file, 0.2 MB. Copyright © 2019 Hershey et al. 2019 Hershey et al. This content is distributed under the terms of the Creative Commons Attribution 4.0 International license.

The abundance of each mutant during the passaging steps was assessed using BarSeq, providing a temporal fitness profile for each gene over the course of the experiment (30). Genes with positive fitness scores reflect mutants with adhesion defects that were enriched in medium that had been depleted by the use of cheesecloth. Genes with negative fitness scores represent hyperadhesive mutants that are depleted more efficiently by cheesecloth than is the wild type. As expected, most genes had inconsequential effects on adhesion. However, there were a significant number of genes whose mutation caused strong cheesecloth-dependent changes in abundance over the course of the multiday experiment. The 250 genes with the highest fitness values (above or below the baseline level) are shown in Fig. 1C. The time-resolved nature of the experiment allowed us to group mutants with similar fitness profiles into distinct classes. Mutants with similar temporal adhesion profiles often mapped to genes with similar or complementary annotations, indicating that they represented groups of functionally related genes. We predicted the functions of uncharacterized genes using known functions for genes with similar fitness profiles. For example, a cluster of genes whose mutants showed a continuous increase in abundance after each passage contained many known *hfs* genes, and any uncharacterized genes that shared this fitness profile would be predicted to contribute to holdfast synthesis as well. We identified four clusters containing mutants that displayed distinct fitness profiles for the cheesecloth passaging experiment. Each cluster is described in detail below.

### Mutants defective in smooth lipopolysaccharide display ectopic adhesion.

**FIG 2:**
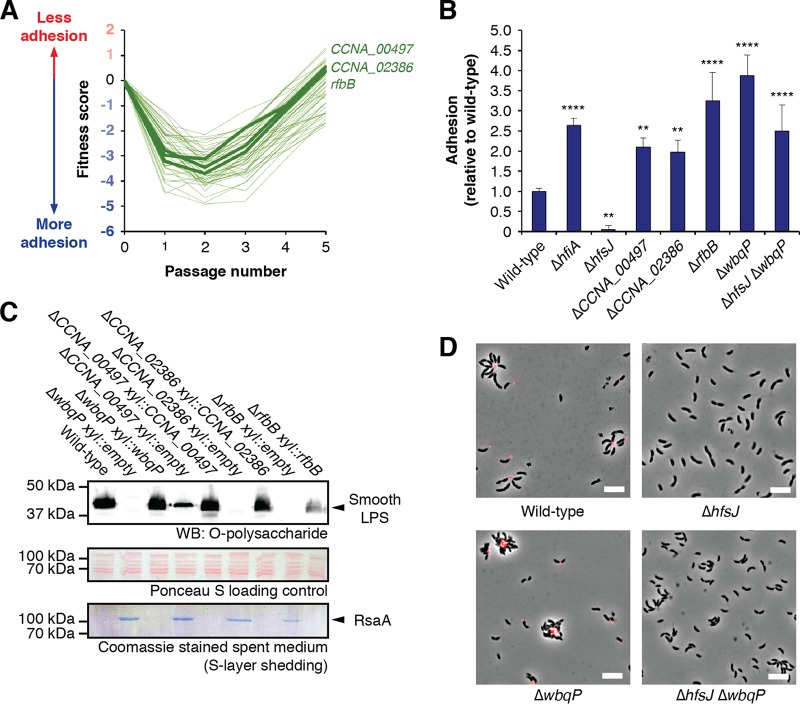
Disrupting SLPS production leads to ectopic adhesion. (A) Fitness profiles for genes in the SLPS cluster. A full list of these genes and their annotations is provided in Table S5. *wbqP* was not characterized in our library due to low insertion density in this region. (B) Surface attachment of SLPS mutants measured by CV staining. Cultures were grown for 24 h in M2X medium before staining of surface-attached cells. Disruption of SLPS led to increased adhesion in a holdfast-independent fashion. The graph shows averages ± standard deviations of results from five biological replicates. Statistical significance was assessed by analysis of variance (ANOVA) with a pairwise Dunnett’s posttest to determine which samples differed from the wild type. **, *P < *0.01; ****, *P < *0.0001. (C) Smooth LPS production was disrupted in mutants Δ*CCNA_00497*, Δ*CCNA_02386*, and Δ*rfbB*. (Top) Western blot (WB) to detect SLPS. (Middle) Total protein stained with Ponceau S as a loading control for SLPS blotting. (Bottom) Coomassie staining of spent medium from the cultures. Each mutant showed a loss of or a decrease in SLPS production by Western blotting and released the S-layer protein RsaA into the spent medium. The cell-surface defects can be complemented by ectopic expression of the appropriate gene. “Empty” refers to plasmid control strains.

We identified a cluster of mutants with strong fitness decreases (i.e., with increased adhesion to cheesecloth) in early passages with relative abundances that recovered to nearly neutral or even positive fitness values as passaging proceeded (Fig. 2A). Many of the genes in this “recovery” cluster had annotations associated with polysaccharide biosynthesis but had no known cellular function. However, the *wbq* genes that are required for the production of smooth lipopolysaccharide (SLPS) comprised a subset of the recovery cluster (31). This suggested that mutants sharing this fitness profile might also be defective in the biosynthesis of SLPS.

10.1128/mBio.02273-18.9 TABLE S5 Fitness scores across cheesecloth passages for mutant clusters shown in Fig. 1C. Fitness values represent averages of data from the three replicates for each passage. The first passage without cheesecloth (PYE1) represents the time 0 sample. Download Table S5, DOCX file, 0.2 MB. Copyright © 2019 Hershey et al. 2019 Hershey et al. This content is distributed under the terms of the Creative Commons Attribution 4.0 International license.

We focused on three uncharacterized genes in the recovery cluster. C. crescentus *CCNA_00497* is annotated as encoding a putative rhamnosyl transferase, *CCNA_02386* is annotated as encoding an O-antigen ligase, and *CCNA_03744* is homologous to *rfbB*, a gene required for the biosynthesis of dTDP-L-rhamnose (32). Mutations in *rfbB* were previously shown to suppress the holdfast attachment defect observed in a Δ*hfaD* mutant, but SLPS was not examined in these mutants (33). We created in-frame deletions of *CCNA_00497*, *CCNA_02386*, and *rfbB* and analyzed SLPS production by immunoblotting. A deletion of *wbqP*, which is thought to encode the initial glycosyltransferase step in the O-polysaccharide biosynthesis pathway, was used as a positive control. Disruption of *CCNA_02386*, *rfbB*, or *wbqP* led to the loss of detectable SLPS, and Δ*CCNA_00497* cells showed a reduction in SLPS levels (Fig. 2C). Additionally, all four mutants released the S-layer protein RsaA into the spent medium, an additional hallmark of SLPS defects in C. crescentus (Fig. 2C) (34). None of the mutants displayed observable changes in rough LPS, demonstrating that they were not defective in the production of lipid A or the core oligosaccharide (see Fig. S1 in the supplemental material). All of the defects could be complemented by ectopic expression of the target gene, confirming their roles in the production of SLPS (Fig. 2C; see also Table S2).

10.1128/mBio.02273-18.1 FIG S1 Analysis of rough LPS in SLPS mutants. The figure shows a silver-stained polyacrylamide gel of rough LPS that was isolated and analyzed as described in Materials and Methods. None of the four SLPS mutants show apparent defects in rough LPS. Download FIG S1, TIF file, 1.1 MB. Copyright © 2019 Hershey et al. 2019 Hershey et al. This content is distributed under the terms of the Creative Commons Attribution 4.0 International license.

10.1128/mBio.02273-18.6 TABLE S2 Complementation of adhesion defects. Normalized crystal violet staining values are shown as averages ± standard deviations of data from at least 4 biological replicates. All values shown reflect trends that were consistent across at least five independent experiments. Cells were grown for 17 h in M2X or for 24 h in PYE medium before staining. n.m., not measured. Download Table S2, DOCX file, 0.1 MB. Copyright © 2019 Hershey et al. 2019 Hershey et al. This content is distributed under the terms of the Creative Commons Attribution 4.0 International license.

The fitness profiles for early stages of cheesecloth passaging suggested that disrupting SLPS led to production of hyperadhesive cells that were rapidly depleted by cheesecloth. In complex medium, nearly all of the wild-type cells produce a holdfast, making the dynamic range for detecting increased adhesion quite small. Thus, we chose to investigate potential hyperadhesive phenotypes by examining mutants using a defined medium in which fewer cells produce a holdfast. We found that the SLPS mutants were indeed hyperadhesive, producing crystal violet (CV) staining values ranging from two to four times the level seen with the wild type (Fig. 2B). To understand the basis of hyperadhesion, Δ*wbqP* cells were imaged after staining with fluorescently labeled wheat germ agglutinin (fWGA) to label holdfasts. Most wild-type cells displayed a fluorescent focus at the tip of the stalk, and stalks from multiple cells often aggregated around a single focus to form the rosette structures that are characteristic of holdfast production. In the Δ*wbqP* background, a comparable number of cells produced a holdfast, but the structure of the rosettes was altered. Cells that assembled around a holdfast were more tightly packed, and not all of them adhered to the aggregates through the tip of the stalk (Fig. 2D).

The unusual rosette structures in the Δ*wbqP* mutant suggested that cells with disrupted SLPS might have a second mode of adhesion that did not require a holdfast. We compared fWGA staining in the holdfast-deficient Δ*hfsJ* strain to fWGA staining of a Δ*hfsJ* Δ*wbqP* double mutant that lacks both holdfast and SLPS. Δ*hfsJ* cells did not stain with fWGA and did not form aggregates. Δ*hfsJ* Δ*wbqP* cells did not stain with fWGA, but, in contrast to the Δ*hfsJ* strain, the cells still formed aggregates (Fig. 2D). These aggregates appeared not to be mediated by stalk-stalk interactions but rather through interactions with the cell body. This further supported the idea that a holdfast-independent mode of ectopic adhesion operates in SLPS mutants. Consistent with this model, bulk adhesion in the Δ*hfsJ* Δ*wbqP* double mutant was not abolished; the level was, in fact, higher than that seen with the wild type (Fig. 2B). We conclude that disrupting SLPS production caused defects in the cell surface leading to a holdfast-independent mode of adhesion that represented the dominant mode of adhesion for these mutants early in our experimental time course.

### The flagellum and type IV pili regulate holdfast production.

A second cluster of mutants primarily contained genes known to participate in chemotaxis and flagellar motility as well as genes corresponding to components of the type IV pilus machinery. The fitness profiles for these mutants suggested that disrupting the assembly of polar appendages, either pili or flagella, leads to hyperadhesion. To study the effects of polar appendages on adhesion, we deleted the genes for the flagellar basal body component FlgH and the pilus assembly protein CpaH (35, 36). We confirmed that the Δ*flgH* mutant showed the expected loss in motility and that the Δ*cpaH* mutant was resistant to the type IV pilus-specific phage ΦCBK (Fig. S2 and S3).

10.1128/mBio.02273-18.2 FIG S2 Validation of pilus and flagellum phenotypes in polar appendage mutants. (Top) Swimming phenotypes for relevant mutants in soft agar. Note that the Δ*pilA* mutant showed a slight but reproducible increase in swarm size that was shown to be able to be complemented by ectopic expression of *pilA* in *trans*. Mutants lacking *flgH* showed the expected nonmotile phenotype. The data are representative of results of four independent experiments. (Bottom) ΦCBK sensitivity of polar appendage mutants. Mutants lacking *pilA* or *cpaH* showed the expected ΦCBK resistance phenotype. The data are representative of results of three independent experiments. Download FIG S2, TIF file, 2.7 MB. Copyright © 2019 Hershey et al. 2019 Hershey et al. This content is distributed under the terms of the Creative Commons Attribution 4.0 International license.

10.1128/mBio.02273-18.3 FIG S3 Comparison of crystal violet staining and holdfast counts for polar appendage mutants. Surface attachment was assessed by CV staining (violet bars) and holdfast production by fWGA staining (orange bars) as described in Materials and Methods. The values corresponding to growth in complex medium are shown on the left and those corresponding to growth in defined medium on the right. Error bars represent standard deviations of data representing results from three independent groups of cells. Download FIG S3, TIF file, 0.8 MB. Copyright © 2019 Hershey et al. 2019 Hershey et al. This content is distributed under the terms of the Creative Commons Attribution 4.0 International license.

**FIG 3:**
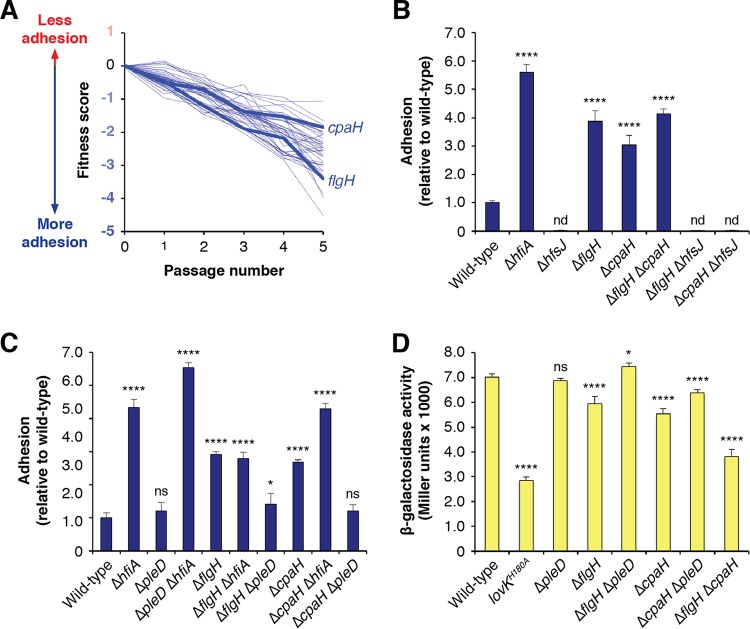
Disrupting polar appendages stimulates holdfast production. (A) Fitness profiles for genes in the polar appendage cluster. A full list of these genes and their annotations is provided in Table S5. (B) Surface attachment of motility mutants measured by CV staining. Cultures were grown for 17 h in M2X medium before staining of surface-attached cells. Deletion of the genes for either the outer membrane flagellar base protein FlgH or the inner membrane type IV pilus component CpaH caused increased adhesion. In both mutant backgrounds, *hfsJ* is required for attachment. The graph shows averages ± standard deviations of results from five biological replicates. (C) Effect of *hfiA* and *pleD* deletions on surface attachment of *cpaH* and *flgH* mutants. The Δ*hfiA* mutation did not affect adhesion in the Δ*flgH* background and increased adhesion in the Δ*cpaH* background. The increased adhesion in both the Δ*flgH* and Δ*cpaH* mutants can be eliminated by deletion of *pleD.* The graph shows averages ± standard deviations of results from four biological replicates. (D) *P_hfiA_-lacZ* reporter activity in polar appendage mutants. The chart shows averages ± standard deviations of results from four biological replicates. Statistical significance was assessed by ANOVA with a pairwise Dunnett’s posttest to determine which samples differed from the wild type. nd, not detected; ns, not significant; *, *P < *0.05; ****, *P < *0.0001. Where appropriate, *P* values for additional pairwise comparisons pertinent to interpretation are indicated in the text.

Both the Δ*flgH* and the Δ*cpaH* mutants showed adhesion defects in complex medium (Table S2). However, in defined medium, both mutants displayed increased adhesion relative to the wild type, indicating that disrupting the pilus or the flagellum causes hyperadhesion under these conditions (Fig. 3B). To reconcile these differences, we used fWGA staining to measure the proportion of cells that produced a holdfast. The Δ*flgH* and Δ*cpaH* mutants produced more holdfasts than the wild type in both complex medium and defined medium (Fig. S3; see also Table S3). We conclude that flagellum and pilus mutations increase holdfast production but that loss of either appendage also leads to holdfast-independent defects in surface colonization. Pili and flagella often have similar effects on surface colonization in other systems (37, 38). Because the baseline level of holdfast production is low in defined medium, the significance of the enhanced holdfast production seen in the Δ*flgH* and Δ*cpaH* backgrounds appears to outweigh that of surface colonization defects under these conditions (Fig. S3).

10.1128/mBio.02273-18.7 TABLE S3 Holdfast counts for polar appendage mutants. The values represent the fraction of cells (corresponding to 1 cell) that stained with a holdfast focus along with the associated standard deviations of data from three biological replicates. The total number of cells counted is shown in parentheses. Cells were harvested from low-density cultures (Materials and Methods) to minimize the formation of rosettes. However, in the event that rosettes were observed, all cells in the rosette were counted as holdfast-producing cells. Download Table S3, DOCX file, 0.06 MB. Copyright © 2019 Hershey et al. 2019 Hershey et al. This content is distributed under the terms of the Creative Commons Attribution 4.0 International license.

A recent report showed that flagellar hook mutants displayed decreased transcription from the *hfiA* promoter (*P_hfiA_*) in defined medium and that this effect did not occur in the absence of the pleiotropic cell cycle regulator PleD (39–41). This led us to examine the relationships between our polar appendage mutants, *hfiA* and *pleD*. We used a *P_hfiA_-lacZ* reporter to measure transcription from the *hfiA* promoter. Because expression from *P_hfiA_* is low in complex medium, the dynamic range for measuring decreased activity is small. Therefore, we focused on transcriptional changes that occurred in defined medium where the baseline activity of *P_hfiA_* was high. The Δ*flgH* and Δ*cpaH* mutants showed reduced *hfiA* transcription in the reporter assay (Fig. 3D). This modest reduction in *P_hfiA_-lacZ* reporter signal was statistically significant, and changes of this magnitude are known to affect holdfast development (11). The decrease in *P_hfiA_-lacZ* signal was abrogated in the Δ*flgH* Δ*pleD* and Δ*cpaH* Δ*pleD* double mutants (Fig. 3D). Likewise, bulk adhesion in defined medium reverted to near wild-type levels in the Δ*flgH* Δ*pleD* and Δ*cpaH* Δ*pleD* mutants, confirming that *pleD* contributed to the modulation of *P_hfiA_* in the pilus and flagellar mutants (Fig. 3C). We note, however, that a full reversion of the hyperadhesive-holdfast phenotype would be predicted to display bulk adhesion levels below that of the wild type due to the holdfast-independent attachment defects seen in pilus and flagellum mutant backgrounds. Thus, while *pleD* did contribute to the enhanced adhesion seen in the Δ*flgH* and Δ*cpaH* mutants, the effect was not completely dependent on the presence of this gene.

To test whether the hyperadhesive phenotypes in the polar appendage mutants could be explained by repression of *hfiA*, we created Δ*flgH* Δ*hfiA* and Δ*cpaH* Δ*hfiA* double mutants. Bulk attachment in the Δ*flgH* Δ*hfiA* strain was not significantly increased relative to the level seen with the Δ*flgH* single deletion (Fig. 3C). This suggests that the Δ*flgH* mutation effectively inactivated the effects of *hfiA* and that holdfast-independent adhesion defects lowered the maximum level of surface attachment that was achievable in a Δ*flgH* background. The enhanced attachment seen in the Δ*cpaH* background was further increased in a Δ*cpaH* Δ*hfiA* double mutant (*P < *0.0001; Fig. 3C). Thus, the Δ*cpaH* mutation had an intermediate effect on *hfiA* activity by dampening but not completely masking its activity. Consistent with this, the fraction of Δ*cpaH* cells that produced a holdfast in defined medium was intermediate between those of the wild-type and Δ*hfiA* mutant strains, supporting the idea that the Δ*cpaH* mutation caused both intermediate enhancement of holdfast production and holdfast-independent surface-attachment defects (Fig. S3; see also Table S3). Finally, the level of bulk adhesion in a Δ*flgH* Δ*cpaH* double mutant was indistinguishable from that seen with the Δ*flgH* mutant, and *P_hfiA_* transcription was lower in the Δ*flgH* Δ*cpaH* mutant than in either the Δ*flgH* single mutant (*P < *0.0001) or the Δ*cpaH* single mutant (*P < *0.0001) (Fig. 3B and C). These results indicate that holdfast production was likely maximized in the Δ*flgH* mutant and that the Δ*flgH* and Δ*cpaH* mutants modulated *P_hfiA_* through separate pathways.

### A complex role for the pilus in regulating adhesion.

**FIG 4:**
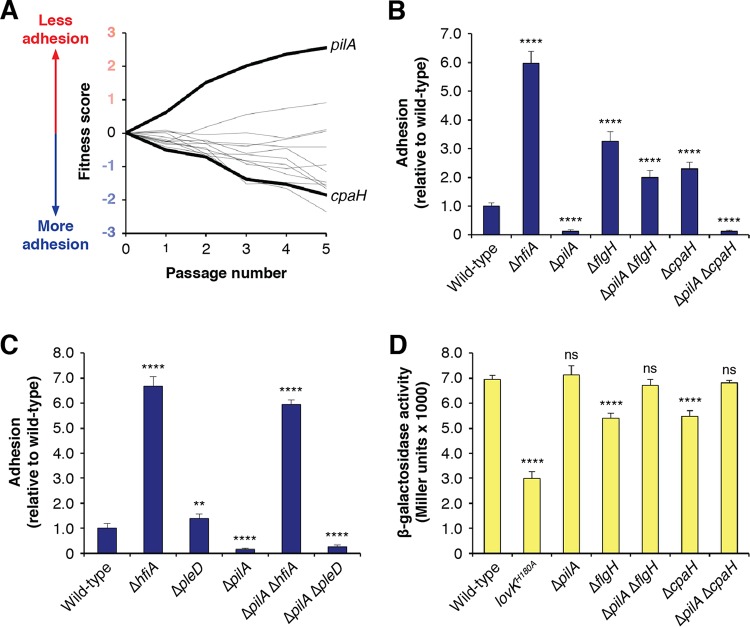
Opposing effects of pilus mutants on adhesion. (A) Fitness profiles for genes at the pilus assembly locus. A full list of these genes and their annotations is provided in Table S5. (B) Surface attachment of pilus mutants measured by CV staining. Cultures were grown for 17 h in M2X medium before staining. Deletion of the gene for the main pilin subunit (PilA) reduced adhesion. The Δ*pilA* mutant was epistatic with respect to the Δ*cpaH* mutant but not to the Δ*flgH* mutant. The graph shows averages ± standard deviations of results from seven biological replicates. (C) Effect of *hfiA* and *pleD* deletions on surface attachment in the Δ*pilA* background. Staining is slightly lower for the Δ*hfiA* Δ*pilA* mutant than for the Δ*hfiA* mutant, reflecting the holdfast-independent defect in surface attachment that occurred when the pilus was disrupted. *pleD* has no effect on adhesion in the Δ*pilA* mutant. The graph shows averages ± standard deviations of results from six biological replicates. (D) *P_hfiA_-lacZ* reporter activity in various *pilA* mutants. The chart shows averages ± standard deviations of results from four biological replicates. Statistical significance was assessed by ANOVA with a pairwise Dunnett’s posttest to determine which samples differed from the wild type. ns, not significant; **, *P < *0.01; ****, *P < *0.0001. Where appropriate, *P* values for additional pairwise comparisons pertinent to interpretation are indicated in the text.

The Δ*cpaH* phenotype suggested that disruption of pilus assembly leads to increased holdfast production via the repression of *hfiA*. However, a closer examination of the fitness profiles for genes involved in type IV pilus assembly revealed a range of phenotypes for various components of the apparatus (Fig. 4A). Most of the genes encoding components of the pilus secretion machinery, including *cpaH*, had fitness profiles consistent with increased adhesion. However, mutations in the gene coding for the main pilin subunit, PilA, displayed the opposite trend. *pilA* mutants had fitness profiles that would be expected for mutants with adhesion defects. We confirmed that, indeed, the Δ*pilA* strain was defective in surface attachment in both complex and defined medium (Fig. 4B; see also Table S2).

To examine the relationship between *pilA-*dependent loss of adhesion and the activation of adhesion observed in mutants that disrupt pilus and flagellum assembly, we created Δ*flgH* Δ*pilA* and Δ*cpaH* Δ*pilA* double mutants. The phenotypes for these mutants were similar in both complex medium and defined medium (Table S4). Surface attachment levels in the Δ*flgH* Δ*pilA* mutant were intermediate with respect to those of the Δ*pilA* mutant (*P < *0.0001) and the Δ*flgH* mutant (*P < *0.0001), suggesting that *flgH* and *pilA* regulate adhesion through independent, additive pathways (Fig. 4C; see also Table S4). In contrast, adhesion in the Δ*cpaH* Δ*pilA* mutant was indistinguishable from that in the Δ*pilA* mutant, demonstrating that the effects of *pilA* on adhesion were epistatic with respect to those of *cpaH* (Fig. 4C; see also Table S4). We conclude that pilin subunit PilA is required for the holdfast-promoting effect caused by disruption of the pilus assembly apparatus.

10.1128/mBio.02273-18.8 TABLE S4 Additional adhesion phenotypes for polar appendage mutants. Normalized crystal violet staining values are shown as averages ± standard deviations of data from at least 4 biological replicates. All values shown reflect trends that were consistent across at least five independent experiments. Cells were grown for 17 h in M2X or 24 h in PYE medium before staining. n.m., not measured. Download Table S4, DOCX file, 0.09 MB. Copyright © 2019 Hershey et al. 2019 Hershey et al. This content is distributed under the terms of the Creative Commons Attribution 4.0 International license.

We further explored the model that *pilA* contributed to the modulation of adhesion by *cpaH* by examining the relationships between *pilA*, *hfiA*, and *pleD*. The decreased adhesion observed in the Δ*pilA* mutant was not affected by the subsequent deletion of *pleD*, indicating that the effect of *pilA* on adhesion is *pleD* independent. Adhesion in a Δ*pilA* Δ*hfiA* double mutant was elevated to a level slightly below that of the Δ*hfiA* strain (*P < *0.0001; Fig. 4B). The difference between the Δ*pilA* Δ*hfiA* and Δ*hfiA* mutants with respect to the levels of surface attachment likely reflects the holdfast-independent surface attachment defects caused by the loss of a functional pilus. Expression from *P_hfiA_* was slightly elevated in the Δ*pilA* mutant (Fig. 4D). However, because *hfiA* was already highly expressed under those conditions, it is difficult to determine if *P_hfiA_* is activated further in the Δ*pilA* mutant. Finally, the β-galactosidase activity shown by the *P_hfiA_* reporter was restored to wild-type levels in both the Δ*flgH* Δ*pilA* and Δ*cpaH* Δ*pilA* backgrounds, demonstrating that *pilA* is required to lower *hfiA* expression in the Δ*flgH* and Δ*cpaH* mutants (Fig. 4D).

### New factors in the holdfast biosynthesis pathway.

The final two clusters of mutants presented in Fig. 1C displayed fitness profiles consistent with adhesion defects. We used the magnitude of the measured fitness changes to separate these genes into (i) a cluster with higher fitness changes that contained all of the *hfs* genes known to be required for robust adhesion and (i) a cluster displaying lower fitness changes which contained the *hfsK* gene. *hfsK* encodes a putative *N*-acyltransferase thought to modify the holdfast polysaccharide in order to produce a fully adhesive holdfast (25). We chose three uncharacterized genes from these clusters of mutants for detailed examination of holdfast defects.

**FIG 5:**
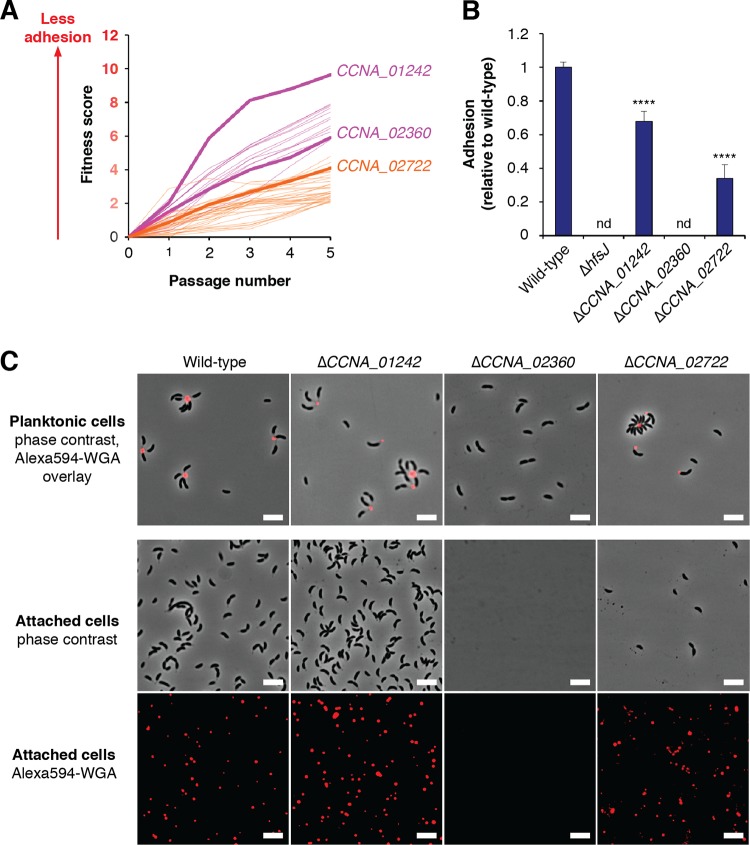
New holdfast biosynthesis factors. (A) Fitness profiles for genes in the *hfs* (magenta) and holdfast modification (orange) clusters. Full lists of these genes and their annotations are provided in Table S5. (B) Surface attachment of putative holdfast mutants measured by CV staining. Cultures were grown for 24 h in PYE medium before being stained. The Δ*CCNA_01242* and Δ*CCNA_02722* mutants displayed reduced staining, and the Δ*CCNA_02360* mutant was nonadhesive. The graph shows averages ± standard deviations of results from five biological replicates. Statistical significance was assessed by ANOVA with a pairwise Dunnett’s posttest to determine which samples differed from the wild type. nd, not detected; ns, not significant; ****, *P < *0.0001. (C) Analysis of holdfast phenotypes by fWGA staining. The top panels show overlays of phase-contrast and fluorescence images after staining of planktonic cells was performed as described in Materials and Methods. Adherent cells from the slide attachment assay are shown as phase-contrast images in the middle set of panels, and the fluorescence channel showing attached holdfast material from the same slides is represented in the bottom panels. The Δ*CCNA_01242* mutant did not have an apparent holdfast defect, the Δ*CCNA_02722* mutant had a holdfast attachment defect, and the Δ*CCNA_02360* mutant did not produce holdfasts. The scale bars represent 5 μm.

Disruption of *CCNA_01242*, which encodes a predicted amino acid permease, led to the strongest nonadhesion fitness profile of any gene in the cheesecloth passaging experiment (Fig. 5A). However, the Δ*CCNA_01242* strain had only a modest defect in surface attachment (Fig. 5B). There were no obvious holdfast defects in the mutant, and we could not detect a significant adhesion defect under any conditions tested (Fig. 5C; see also Table S2). Instead, Δ*CCNA_01242* had an unusual, biphasic growth profile. In complex medium, the log phase was shorter than that shown by the wild type, leading to a lower level of optical density (OD) as growth began to slow prematurely. Growth of this strain continued slowly over the next 24 h and eventually plateaued at an OD similar to that seen with the wild type (Fig. S4). Such biphasic growth seems to confound fitness calculations for samples collected during the sequential passaging experiment. It is not clear why *CCNA_01242* mutants were more enriched when cheesecloth was included in the medium, but we conclude nonetheless that this gene does not contribute to holdfast production.

10.1128/mBio.02273-18.4 FIG S4 Complementation of growth defect in the ΔCCNA_01242 mutant. The ΔCCNA_01242 mutant displayed a biphasic growth curve indicating a defect in later growth phases. Normal growth was restored by ectopic complementation with *CCNA_01242.* Download FIG S4, TIF file, 0.4 MB. Copyright © 2019 Hershey et al. 2019 Hershey et al. This content is distributed under the terms of the Creative Commons Attribution 4.0 International license.

*CCNA_02360* is a predicted member of the GT2 family of glycosyltransferases. Its disruptions have a fitness profile closely resembling the profiles of many of the known *hfs* genes (Fig. 5A). A Δ*CCNA_02360* mutant was nonadhesive in surface attachment assays and did not stain with fWGA under any conditions tested (Fig. 5B and C). Given the lack of holdfast production in Δ*CCNA_02360* cells, we predict that *CCNA_02360* encodes a glycosyltransferase that contributes one or more monosaccharides to repeating unit of the holdfast polysaccharide and have named this gene *hfsL.* Previous studies have described mutants in *CCNA_02360* as holdfast-deficient control strains (25, 40), but, curiously, identification of the gene and characterization of its phenotype have not been reported. Closely related genes could be identified in many stalked bacteria within the *Caulobacterales* clade, suggesting that HfsL carries out a conserved step in holdfast biosynthesis. Identifying close homologs in more distantly related *Alphaproteobacteria* was difficult due to the abundance of GT2 family glycosyltransferases in bacterial genomes. Nevertheless, HfsL represents a fourth glycosyltransferase that is required for holdfast biosynthesis in C. crescentus.

*CCNA_02722* encodes a hypothetical protein that does not show homology to any known protein families. It has a predicted N-terminal signal peptide for export to the periplasm. The fitness profile is similar to that of *hfsK*, which is indicative of modest defects in adhesion (Fig. 5A). The Δ*CCNA_02722* mutant was significantly impaired in surface-attachment (Fig. 5B). When planktonic Δ*CCNA_02722* cells were stained with fWGA, holdfast staining was apparent (Fig. 5C). However, using adhesion assays in which cells were grown in the presence of a glass slide that was then washed, stained with fWGA, and imaged, we detected a holdfast anchoring defect. Wild-type cells normally coat the surface of the slide and show fWGA foci at the site of attachment, but we observed very few attached Δ*CCNA_02722* cells. Instead, the slide was coated with an abundance of fWGA-reactive material (Fig. 5C). This holdfast shedding phenotype is characteristic of mutants with defects in anchoring the holdfast matrix to the surface of the cell (28), and the gene has been named *hfaE* accordingly. Like *hfsL*, *hfaE* could be identified in the genomes of many other stalked bacteria, suggesting a conserved role in holdfast anchoring.

## DISCUSSION

Holdfast production in C. crescentus presents an attractive model system to interrogate the assembly of polysaccharides in bacteria. Lectin staining, enzymatic sensitivity, and functional annotations for the *hfs* genes indicate that the holdfast contains a polysaccharide (17, 18, 23). However, despite decades of work, surprisingly little else is known about the chemical structure of the holdfast. As part of our efforts to characterize the biosynthetic pathway, we sought a complete list of enzymes required for holdfast biosynthesis. In order to saturate the search for *hfs* genes, we developed a TnSeq-based method to measure the adhesion phenotype conferred by each nonessential gene in the genome.

The genome-wide approach provided a surprisingly rich set of insights into adhesion in C. crescentus. Not only did the screen identify nonadhesive mutants representing missing components of the holdfast pathway, but it also resolved mutants that displayed increased adhesion. One class of such mutants was defective in the production of smooth LPS. Disrupting SLPS resulted in elevated adhesion levels in both holdfast-producing and holdfast-deficient backgrounds. SLPS mutants no longer adhered exclusively at the stalks of holdfast-producing cells but instead displayed a generalized form of adhesion throughout the cell surface. Our analysis of these mutants supports a model in which the C. crescentus envelope is structured to ensure that the cell surface is nonadhesive, maximizing the opportunity for polar adhesion via the holdfast. It is still unclear why disrupting the cell surface led to the depletion and recovery profile seen during cheesecloth passaging. Regardless of the mechanism, simply identifying mutants that shared this temporal profile allowed the characterization of new SLPS biosynthesis genes. Such cofitness approaches have been useful in other contexts (42) and allowed us to greatly expand the number of genes identified in the C. crescentus SLPS pathway (see Table S5 in the supplemental material).

Genes with predicted functions in motility, flagellar biosynthesis, and type IV pilus assembly displayed a hyperadhesive profile that could be distinguished from the profile of SLPS mutants by the lack of a recovery phase. Mutations in components of the flagellar basal body were recently shown to enhance holdfast production by inhibiting the expression of *hfiA*, a result that we confirmed here in our examination of *flgH* (41). Cofitness analysis indicated that the *cpa* genes, which code for components of the type IV pilus, have a similar phenotype, and we showed that mutation of the inner membrane pilus assembly component gene *cpaH* also increased holdfast production by repressing *hfiA*. However, mutation of *pilA*, which codes for the main subunit of the pilus filament, reduced adhesion. The adhesion defect in Δ*pilA* cells was partially restored in a Δ*pilA* Δ*flgH* background but remained unchanged in Δ*pilA* Δ*cpaH* cells. Thus, although both the Δ*flgH* and Δ*cpaH* mutations enhanced holdfast production, the two pathways can be distinguished by their requirement for *pilA*. Disentangling the specific routes by which the various polar appendage mutants modulate *hfiA* activity will require identifying intermediate factors in the signaling pathways, but our results underscore the interconnectedness of the flagellum, pilus, and holdfast.

Numerous reports have debated the roles of pili and the flagellum in surface attachment (13–15, 43, 44). Our unbiased, genome-wide analysis of adhesion unambiguously identified both appendages as determinants of attachment. Two recent studies, in particular, showed that mutating the flagellar basal body represses *hfiA* and that disruption of flagellar rotation upon surface contact stimulates holdfast production (15, 41). In our cheesecloth passaging experiment, the flagellar motor (*mot*), flagellin glycosylation (*flm*), and chemotaxis (*che*) genes shared the same hyperadhesive fitness profile as components of the flagellar basal body (Table S5). Some of these mutants would be predicted to disrupt flagellar rotation without affecting assembly *per se* (45). We propose that disrupting flagellar function, either through physical interaction with a surface or through mutation of motility genes, stimulates holdfast production. It will be interesting to test this model by determining whether the repression of *hfiA* seen in the flagellar mutants is required to activate holdfast production after surface contact. We also note that future studies should take into account the finding that mutating *flgH* reduces biofilm formation in a holdfast-independent manner (see Fig. S3 in the supplemental material), suggesting that flagellar motility likely also promotes productive interactions with a surface that lead to permanent attachment.

Much like flagellar mutants, disrupting components of the type IV pilus causes both increased holdfast production and holdfast-independent surface colonization defects. A recent report proposed that contact with a surface inhibits the retraction of PilA filaments, leading to a stimulation of holdfast production (14). One might initially conclude that pilus assembly defects in the *cpa* mutants mimic the obstruction of pilus filament retraction. However, the situation is more complex because *pilA* was required for increased holdfast production in the Δ*cpaH* mutant. These findings can be reconciled in a model in which the disruption of filament oscillation caused either by surface contact or upon mutation of the *cpa* genes leads to increases in the pool of unassembled PilA proteins that serve as a signal to stimulate holdfast production. A similar model was proposed for the regulation of biofilm formation by the Agrobacterium tumefaciens pilus (38). Furthermore, unassembled pilin subunits in Pseudomonas aeruginosa directly activate the sensor kinase PilS, leading to the repression of *pilA* transcription (46). Although no clear PilS homologs are found in C. crescentus, this example demonstrates that the membrane-associated pilin pool can serve as an input that activates signaling cascades.

An important aspect of both the flagellar and pilus pathways for holdfast regulation is their partial dependence on *pleD*. *pleD* is a pleiotropic regulator of cell polarity that is required for flagellar ejection and stalk synthesis during the swarmer-cell-to-stalked-cell transition (47). It functions as a diguanylate cyclase that is activated by phosphorylation at distinct stages of the cell cycle to produce the bacterial second messenger cyclic di-GMP (cdG) (39, 48). Though *pleD* has been shown to regulate the timing of holdfast synthesis during the cell cycle, Δ*pleD* mutants do not have significant bulk adhesion defects (Fig. 3C) (40). More broadly, placement of *pleD* within a signaling cascade that regulates holdfast synthesis is confounded by the fact that both *pleD* and cdG contribute to numerous processes that intersect with holdfast synthesis, including flagellar function, pilus assembly, stalk biogenesis, and cell cycle progression (39, 49). Importantly, it has been shown that the holdfast glycosyltransferase HfsJ, which is the target of inhibition by HfiA, is also directly activated by cdG (11, 15). Thus, the roles of PleD in promoting holdfast synthesis upon disruption of the pilus or flagellum are likely twofold. It activates HfsJ by producing cdG and relieves inhibition by lowering transcription of *hfiA* through an unknown mechanism. PleD-dependent increases in cdG concentrations may account for the disparity between the large changes in adhesion and the modest decreases in *hfiA* expression in the Δ*flgH* and Δ*cpaH* backgrounds (Fig. 3).

**FIG 6:**
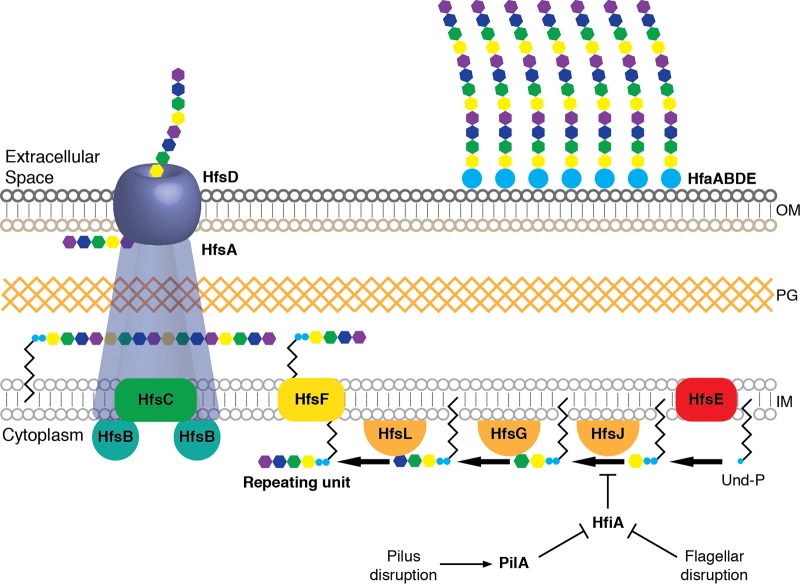
Updated model for holdfast biosynthesis. The model shows a *wzy-*type polysaccharide biosynthesis pathway. Four glycosyltransferases, HfsE, HfsJ, HfsG, and newly described HfsL, add monsaccharides sequentially onto the UPP carrier to produce a glycolipid repeating unit. This intermediate is flipped across the membrane by HfsF, polymerized by HfsC, and exported by a putative HfsABD transevelope complex. Attachment of the holdfast matrix is mediated by the Hfa proteins, including newly identified HfaE, reported here. Disruptions to the flagellum or the pilus activate holdfast production by relieving the inhibition of HfsJ by HfiA. OM, outer membrane; PG, peptidoglycan; IM, inner membrane.

Finally, the initial goal of this study was to saturate the search for holdfast production factors. We used the cheesecloth passages to define a temporal fitness profile that was shared by genes known to be required for holdfast biosynthesis and used this pattern to search for missing genes in the pathway. In addition to a new holdfast anchoring factor, *hfaE*, we identified a glycosyltransferase, *hfsL*, and showed that it is required for holdfast production. Due to the saturating nature of our experiment, we believe that HfsE (along with the redundant PssY and PssZ), HfsJ, HfsG, and HfsL carry out the only glycosyltransferase steps required for holdfast biosynthesis (Fig. 6). Thus, we predict that a repeating unit of four or more monosaccharides makes up the holdfast polysaccharide because each of these enzymes likely contributes at least one sugar to the glycolipid intermediate that serves as a substrate for polymerization.

Implicit in defining the complete set of genes in the holdfast pathway is knowledge of genes that do not contribute. Many bacterial polysaccharides contain specialized monosaccharide components (50, 51), and these intermediates are often synthesized as nucleotide-activated precursors that are directly utilized by glycosyltransferases (52, 53). We did not identify any genes involved in nucleotide sugar metabolism that had significant adhesion defects when disrupted. The modification factors HfsH and HfsK do likely convert certain monosaccharide components into more specialized residues. However, functional annotations for these enzymes predict that they act on lipid-linked or polymerized substrates downstream of the glycosyltransferases, and neither enzyme is explicitly required for holdfast synthesis (19, 25). Our results suggest that specialized sugar precursors are not needed to produce the holdfast and that it is instead built using “housekeeping” sugars that are shared by other cellular processes. The ability to utilize standard nucleotide sugars as substrates without a requirement for specialized chemical syntheses will make the holdfast biosynthetic enzymes useful models for probing the catalytic mechanisms of bacterial polysaccharide biosynthesis.

The findings reported here highlight the advantages of probing mutant phenotypes in parallel. Classical genetic selection methods in which mutants are enriched and then isolated and analyzed separately inherently favor the most extreme phenotypes. The single-strain approach also favors longer genes that contribute more individual mutants to the pool. Screening mutants in parallel using TnSeq reduces these biases, allowing detection of a range of phenotypes and capturing phenotypes for less-abundant mutants in the pool. In this study, we discovered new regulatory networks that modulate holdfast synthesis by identifying mutants that display modest adhesion increases under conditions in which cells are already adhesive. Such mutants would be extremely difficult to isolate using a classical approach. Additionally, the saturating nature of these experiments makes them ideal for outlining complete biosynthetic pathways. Having a reasonable measure of saturation allowed us to leverage the results of the genome-wide screen to propose a model for the enzymatic steps in the holdfast pathway that will inform efforts to reconstitute the biosynthetic reactions *in vitro* (Fig. 6).

## MATERIALS AND METHODS

### Strains, growth conditions, and genetic manipulation.

Strains and plasmids used in this study are listed in Table S6 in the supplemental material. Escherichia coli strains were grown in LB at 37°C with diaminopimelic acid (60 mM), kanamycin (50 μg/ml), or tetracycline (12 μg/ml) included as needed. C. crescentus CB15 was grown in either peptone-yeast extract (PYE; complex) medium or M2 salts containing 0.15% xylose (M2X, defined) at 30°C (54). When necessary, sucrose (3%), kanamycin (25 μg/ml), or tetracycline (2 μg/ml) was included in solid medium. Kanamycin (5 μg/ml) or tetracycline (1 μg/ml) was included in liquid medium when necessary. Standard techniques were used for Gibson assembly-based cloning and sequencing plasmids (55). The primer sequences used for cloning specific constructs are available on request. Plasmids were introduced by electroporation or, in the case of the pFC1948 reporter plasmid, by triparental mating. Unmarked mutations were created through two-step deletion using SacB counterselection. Mutants were complemented by insertion of target genes into pXGFPC-2, which allows the integration of the plasmid at the *xyl* locus and transcriptional control from *P_xyl_* (56). When necessary, target genes were inserted into pXGFPC-2 in reverse orientation under the control of their native promoters.

10.1128/mBio.02273-18.10 TABLE S6 Strains and plasmids used in this study. To analyze the sequence data used for fitness calculations, we used the C. crescentus NA1000 genome annotations. NA1000 is directly derived from CB15, but its genome has more-detailed annotations and is better curated. To facilitate interpretation of the BarSeq data, the genotypes described in the text and supplemental tables used the NA1000 locus numbers and nomenclature. We note the corresponding CB15 locus numbers in the “Description” column. Download Table S6, DOCX file, 0.2 MB. Copyright © 2019 Hershey et al. 2019 Hershey et al. This content is distributed under the terms of the Creative Commons Attribution 4.0 International license.

### Library development and mapping.

The barcoded HiMar transposon pool APA_752, developed by Wetmore et al. (30), was used to create a barcoded Tn library in C. crescentus CB15. Construction of the library was reported previously, along with its associated statistics (57). Briefly, the transposon pool was introduced into C. crescentus by conjugation. Transconjugants appearing on selective plates were pooled, used to inoculate a liquid culture with kanamycin, and grown for 3 doublings. Glycerol was added to achieve a final concentration of 15%, and 1 ml aliquots were frozen and stored at −80°C. TnSeq also followed the method of Wetmore et al. (30). A 1-ml library aliquot was centrifuged, and genomic DNA was extracted from the pellet. The DNA was sheared, size selected for ∼300-bp fragments, and end repaired. A custom Y-adapter (Mod2_TS_Univ annealed to Mod2_TruSeq) was ligated, and transposon junctions were amplified by PCR using the Nspacer_BarSeq_pHIMAR and P7_mod_TS_index1 primers. An Illumina HiSeq 2500 system was used to generate 150-bp single-end reads of the library. The genomic positions of each barcoded insertion were determined with BLAT. The barcode corresponding to each insertion site was determined using MapTnSeq.pl. This information was used to develop a list of barcodes that mapped to unique insertion sites using DesignRandomPool.pl (available at https://bitbucket.org/berkeleylab/feba).

### Passaging in cheesecloth.

Aliquots (1 ml) of the transposon library were thawed at room temperature, a 300-μl volume of the library was inoculated into a well of a 12-well microtiter plate containing 1.2 ml PYE, and a stack of 5 squares (∼10 mm by ∼10 mm) of sterile cheesecloth was added. The culture was grown with shaking at 155 rpm at 30°C for 24 h, after which 100 μl of the planktonic culture was used to inoculate a fresh well containing 1.4 ml PYE and a fresh piece of sterile cheesecloth. An additional 500 μl of the planktonic culture medium was centrifuged, and the pellet was stored at −20°C for BarSeq analysis. The process was repeated for a total of five passages, and each passaging experiment was performed in triplicate. The same procedure was used to perform passaging experiments in which no cheesecloth was added.

### Fitness determination with BarSeq.

Cell pellets from 0.5 ml of planktonic culture medium that had been frozen after each passage were used as templates for PCRs that simultaneously amplified the barcode region of the transposon insertions and added TruSeq indexed Illumina adaptors (30). The PCR products were purified, pooled, and multiplexed on a single Illumina 4000 lane for sequencing. Fitness values for each gene were determined using the pipeline described by Wetmore et al. (30). Barcodes for each read were mapped using MultiCodes.pl and correlated with their associated insertion positions using combineBarSeq.pl. The data were used to calculate fitness using FEBA.R. This analysis determines strain fitness as the log_2_ ratio of barcode counts in a sample to the barcode counts determined under the reference conditions, for which we used the first passage of the library in PYE without cheesecloth. Gene fitness was calculated by determining the weighted average of the insertion mutants with a given gene, excluding the first and last 10% of the open reading frame (ORF). The scripts used for fitness determination can be found at https://bitbucket.org/berkeleylab/feba.

### Analysis of fitness data.

We focused on identifying the genes with the highest absolute (positive or negative) fitness scores. For each passaging step (with and without cheesecloth), an average and a standard deviation for the three replicate samples were calculated. Genes for which the largest standard deviation across the 10 conditions was greater than the largest absolute fitness score were eliminated from further analysis. Fitness scores determined for each passage performed without cheesecloth were subtracted from those for the corresponding passage performed with cheesecloth to normalize for growth defects. These normalized values were used to rank each gene according to its largest absolute fitness score at any stage of cheesecloth passaging. The top 250 genes were then sorted by hierarchical clustering (58) to identify related fitness patterns. Groups derived from the clustering analysis were curated manually to produce the gene data shown in Fig. 1C. The “Pilus Assembly” subsection of Table S5 was created by manually identifying known pilus assembly genes, some of which do not have phenotypes that are strong enough to meet the cutoff used to identify the genes in Fig. 1C.

### Crystal violet (CV) staining of adherent cells.

Overnight cultures of C. crescentus grown in PYE were diluted to an OD at 600 nm (OD_660_) of 0.4 with PYE, 1 μl was inoculated into the wells of 48-well microtiter plates containing 450 μl medium, and the cultures were shaken for 24 h. For the cultures used to measure the effects of polar appendage mutants on adhesion, the incubation was shortened to 17 h. The cultures were then discarded, and the plates were washed thoroughly with a steady stream of tap water. The surface-attached cells remaining in the wells were stained for 5 min with 0.01% crystal violet and washed again with tap water. Stain was extracted for 5 min in 100% ethanol (EtOH) and quantified by reading absorbance at 575 nm.

### Fluorescent wheat germ agglutinin (fWGA) staining.

For staining of planktonic cells, 400 μl of liquid culture was added to an Eppendorf tube and Alexa594-conjugated WGA (Thermo Fisher) was added to achieve a final concentration of 0.5 μg/ml. After incubation of the cells for 5 min in the dark, 1 ml of sterile water was added and the cells were centrifuged at 6,000 × *g* for 2 min. The pellet was resuspended in 5 μl of medium, spotted on a glass slide, and imaged. Cells were imaged with a Leica DM500 microscope equipped with a HCX PL APO 63×/1.4-numerical-aperture (NA) Ph3 objective. fWGA staining was visualized using a red fluorescent protein (RFP) fluorescence filter (Chroma set 41043). For quantifying the number of holdfast-producing cells, a culture was inoculated to reach an OD_660_ of 0.002 and harvested when the OD_660_ reached 0.05 to 0.1 to minimize the number of rosettes.

For staining attached cells, a glass coverslip that had been washed with ethanol was added to 1 ml of PYE in a 12-well plate, and 100 μl of a starter culture (diluted to an OD_660_ of 0.4) was added to the well. The cultures were grown for 6 to 8 h. Nonattached cells were removed from the coverslip by washing under a stream of distilled water. One side of the glass was covered with a solution of 0.5 μg/ml fWGA–PYE, incubated for 5 min in the dark, and washed under a stream of distilled water. The coverslip was placed stain side down on a glass slide and imaged with phase contrast and fluorescence as described above.

### Smooth lipopolysaccharide (SLPS) immunoblotting.

Lysates were prepared for immunoblotting by the method of Walker et al. (59). Pellets from saturated cultures were treated with DNase and lysozyme, mixed with SDS-running buffer, and digested with proteinase K. Samples were separated on a Tris-glycine SDS-PAGE gel with 12% acrylamide and transferred to nitrocellulose. The amount of sample loaded was normalized to the final optical density of the culture at harvest. The membrane was blocked in 5% milk, probed with a 1-in-20,000 dilution of anti-SLPS serum raised in rabbit (34), washed, probed with horseradish peroxidase (HRP)-conjugated goat anti-rabbit antibody (Invitrogen), washed again, and visualized with peroxidase substrate. The top half of the membrane was removed before blocking and stained with Ponceau S (0.25% [wt/vol] Ponceau S–1% acetic acid) as a loading control.

### Analysis of rough LPS.

LPS was extracted by the method of Darveau and Hancock (60). Cells were isolated from saturated 50-ml C. crescentus cultures grown in M2X by centrifugation, resuspended in 2 ml of 10 mM Tris-HCl (pH 8.0) containing 2 mM MgCl_2_, and sonicated. DNase I and RNase A were added to achieve concentrations of 100 μg/ml and 25 μg/ml, respectively, and the lysate was incubated for 1 h at 37°C. Additional DNase I and RNase A were added to achieve concentrations of 200 μg/ml and 50 μg/ml, respectively, and the lysate was incubated for an additional 1 h at 37°C. SDS and EDTA were added to achieve concentrations of 2% and 100 mM, respectively, and the lysate was incubated for 2 h at 37°C. The solution was then centrifuged for 30 min at 50,000 × *g*. Proteinase K was added to achieve a concentration of 50 μg/ml in the supernatant, and the solution was incubated for 2 h at 60°C, after which the LPS was precipitated with 2 volumes of ice-cold 0.375 M MgCl_2_–95% EtOH and collected by centrifugation at 12,000 × *g*. The precipitate was resuspended in 2% SDS containing 100 mM EDTA, incubated overnight at 37°C, and reprecipitated with 0.375 M MgCl_2_–95% EtOH. The precipitate was then suspended in 10 mM Tris-HCl (pH 8.0) and centrifuged for 2 h at 200,000 × *g*. The LPS pellets from each strain were suspended in SDS loading dye and separated by Tris-Tricine SDS-PAGE on an 18% acrylamide gel containing 6 M urea. LPS was stained using the periodate-silver method of Kittelberger and Hilbink (61).

### Bacteriophage ΦCBK sensitivity.

Saturated cultures of C. crescentus grown in PYE were diluted to an OD_660_ of 0.4 with PYE. Volumes of 4 μl of these suspensions, along with six 10-fold serial dilutions (prepared in PYE), were spotted on PYE plates containing 0.15% xylose that had been spread to 10^7^ PFU/ml (assuming a volume of 60 ml for each 150-mm-diameter plate) with ΦCBK or on PYE plates with 0.15% xylose alone. The plates were incubated for 48 h at 30°C and photographed.

### Soft-agar swarming assay.

A 1.5-μl volume from a saturated culture of the appropriate C. crescentus strain grown in PYE was spotted in plates of PYE containing 0.3% agar and 0.15% xylose. The plates were incubated for 4 days at 30°C and photographed.

### *lacZ* reporter assay.

Cultures for measuring reporter activity were grown in M2X medium, and the amount of culture required to achieve an OD_660_ of 0.0005 to 0.00075 was added to fresh M2X medium. These cultures were grown to an OD_660_ of 0.05 to 0.15, and the β-galactosidase activity was measured as previously described (11, 62).

### Data availability.

The raw sequence data for each BarSeq sample and a table describing the barcode abundances and their genomic insertion sites have been uploaded to the NCBI Gene Expression Omnibus (GEO) at https://www.ncbi.nlm.nih.gov/geo/ under accession no. GSE119738.

## References

[1] Silhavy TJ, Kahne D, Walker S 2010 The bacterial cell envelope. Cold Spring Harb Perspect Biol 2:a000414. doi:10.1101/cshperspect.a000414. 20452953 PMC2857177

[2] Whitfield C 2006 Biosynthesis and assembly of capsular polysaccharides in Escherichia coli. Annu Rev Biochem 75:39–68. doi:10.1146/annurev.biochem.75.103004.142545. 16756484

[3] Joiner KA 1988 Complement evasion by bacteria and parasites. Annu Rev Microbiol 42:201–230. doi:10.1146/annurev.mi.42.100188.001221. 3059994

[4] Roberson EB, Firestone MK 1992 Relationship between desiccation and exopolysaccharide production in a soil Pseudomonas sp. Appl Environ Microbiol 58:1284–1291. 16348695 10.1128/aem.58.4.1284-1291.1992 PMC195588

[5] Geno KA, Gilbert GL, Song JY, Skovsted IC, Klugman KP, Jones C, Konradsen HB, Nahm MH 2015 Pneumococcal capsules and their types: past, present, and future. Clin Microbiol Rev 28:871–899. doi:10.1128/CMR.00024-15. 26085553 PMC4475641

[6] Whitfield C 2010 Polymerases: glycan chain-length control. Nat Chem Biol 6:403–404. doi:10.1038/nchembio.376. 20479750

[7] Poindexter JS 1964 Biological properties and classification of the *Caulobacter* group. Bacteriol Rev 28:231–295. 14220656 10.1128/br.28.3.231-295.1964 PMC441226

[8] Degnen ST, Newton A 1972 Chromosome replication during development in Caulobacter crescentus. J Mol Biol 64:671–680. 5022192 10.1016/0022-2836(72)90090-3

[9] Henrici AT, Johnson DE 1935 Studies of freshwater bacteria: II. Stalked bacteria, a new order of Schizomycetes. J Bacteriol 30:61–93. 16559821 10.1128/jb.30.1.61-93.1935 PMC543637

[10] Tsang PH, Li G, Brun YV, Freund LB, Tang JX 2006 Adhesion of single bacterial cells in the micronewton range. Proc Natl Acad Sci U S A 103:5764–5768. doi:10.1073/pnas.0601705103. 16585522 PMC1458647

[11] Fiebig A, Herrou J, Fumeaux C, Radhakrishnan SK, Viollier PH, Crosson S 2014 A cell cycle and nutritional checkpoint controlling bacterial surface adhesion. PLoS Genet 10:e1004101-14. doi:10.1371/journal.pgen.1004101. 24465221 PMC3900383

[12] Purcell EB, Siegal-Gaskins D, Rawling DC, Fiebig A, Crosson S 2007 A photosensory two-component system regulates bacterial cell attachment. Proc Natl Acad Sci U S A 104:18241–18246. doi:10.1073/pnas.0705887104. 17986614 PMC2084327

[13] Li G, Brown PJB, Tang JX, Xu J, Quardokus EM, Fuqua C, Brun YV 2012 Surface contact stimulates the just-in-time deployment of bacterial adhesins. Mol Microbiol 83:41–51. doi:10.1111/j.1365-2958.2011.07909.x. 22053824 PMC3245333

[14] Ellison CK, Kan J, Dillard RS, Kysela DT, Ducret A, Berne C, Hampton CM, Ke Z, Wright ER, Biais N, Dalia AB, Brun YV 2017 Obstruction of pilus retraction stimulates bacterial surface sensing. Science 358:535–538. doi:10.1126/science.aan5706. 29074778 PMC5805138

[15] Hug I, Deshpande S, Sprecher KS, Pfohl T, Jenal U 2017 Second messenger-mediated tactile response by a bacterial rotary motor. Science 358:531–534. doi:10.1126/science.aan5353. 29074777

[16] Kurtz HD Jr, Smit J 1992 Analysis of a Caulobacter crescentus gene cluster involved in attachment of the holdfast to the cell. J Bacteriol 174:687–694. 1732204 10.1128/jb.174.3.687-694.1992 PMC206144

[17] Smith CS, Hinz A, Bodenmiller D, Larson DE, Brun YV 2003 Identification of genes required for synthesis of the adhesive holdfast in Caulobacter crescentus. J Bacteriol 185:1432–1442. 12562815 10.1128/JB.185.4.1432-1442.2003 PMC142846

[18] Toh E, Kurtz HD, Brun YV 2008 Characterization of the Caulobacter crescentus holdfast polysaccharide biosynthesis pathway reveals significant redundancy in the initiating glycosyltransferase and polymerase steps. J Bacteriol 190:7219–7231. doi:10.1128/JB.01003-08. 18757530 PMC2580695

[19] Wan Z, Brown PJB, Elliott EN, Brun YV 2013 The adhesive and cohesive properties of a bacterial polysaccharide adhesin are modulated by a deacetylase. Mol Microbiol 88:486–500. doi:10.1111/mmi.12199. 23517529 PMC3633684

[20] Samuel G, Reeves P 2003 Biosynthesis of O-antigens: genes and pathways involved in nucleotide sugar precursor synthesis and O-antigen assembly. Carbohydr Res 338:2503–2519. 14670712 10.1016/j.carres.2003.07.009

[21] Islam ST, Lam JS 2014 Synthesis of bacterial polysaccharides via the Wzx/Wzy-dependent pathway. Can J Microbiol 60:697–716. doi:10.1139/cjm-2014-0595. 25358682

[22] Hong Y, Reeves PR 2014 Diversity of O-antigen repeat unit structures can account for the substantial sequence variation of Wzx translocases. J Bacteriol 196:1713–1722. doi:10.1128/JB.01323-13. 24532778 PMC3993327

[23] Merker RI, Smit J 1988 Characterization of the adhesive holdfast of marine and freshwater caulobacters. Appl Environ Microbiol 54:2078–2085. 16347718 10.1128/aem.54.8.2078-2085.1988 PMC202806

[24] Hernando-Pérez M, Setayeshgar S, Hou Y, Temam R, Brun YV, Dragnea B, Berne C 2018 Layered structure and complex mechanochemistry underlie strength and versatility in a bacterial adhesive. mBio 9:e02359-17. doi:10.1128/mBio.02359-17. PMC5801468 29437925

[25] Sprecher KS, Hug I, Nesper J, Potthoff E, Mahi M-A, Sangermani M, Kaever V, Schwede T, Vorholt J, Jenal U 2017 Cohesive properties of the *Caulobacter crescentus* holdfast adhesin are regulated by a novel c-di-GMP effector protein. mBio 8:e00294-17. doi:10.1128/mBio.00294-17. 28325767 PMC5362036

[26] Berne C, Ma X, Licata NA, Neves BRA, Setayeshgar S, Brun YV, Dragnea B 2013 Physiochemical properties of Caulobacter crescentus holdfast: a localized bacterial adhesive. J Phys Chem B 117:10492–10503. doi:10.1021/jp405802e. 23924278 PMC3926197

[27] Umbreit TH, Pate JL 1978 Characterization of the holdfast region of wild-type cells and holdfast mutants of Asticcacaulis biprosthecum. Arch Microbiol 118:157–168. doi:10.1007/BF00415724.

[28] Ong CJ, Wong ML, Smit J 1990 Attachment of the adhesive holdfast organelle to the cellular stalk of *Caulobacter crescentus*. J Bacteriol 172:1448–1456. 2307655 10.1128/jb.172.3.1448-1456.1990 PMC208619

[29] van Opijnen T, Bodi KL, Camilli A 2009 Tn-seq: high-throughput parallel sequencing for fitness and genetic interaction studies in microorganisms. Nat Methods 6:767–772. doi:10.1038/nmeth.1377. 19767758 PMC2957483

[30] Wetmore KM, Price MN, Waters RJ, Lamson JS, He J, Hoover CA, Blow MJ, Bristow J, Butland G, Arkin AP, Deutschbauer A 2015 Rapid quantification of mutant fitness in diverse bacteria by sequencing randomly bar-coded transposons. mBio 6:e00306-15. doi:10.1128/mBio.00306-15. 25968644 PMC4436071

[31] Awram P, Smit J 2001 Identification of lipopolysaccharide O antigen synthesis genes required for attachment of the S-layer of Caulobacter crescentus. Microbiology 147:1451–1460. doi:10.1099/00221287-147-6-1451. 11390676

[32] Jiang XM, Neal B, Santiago F, Lee SJ, Romana LK, Reeves PR 1991 Structure and sequence of the rfb (O antigen) gene cluster of Salmonella serovar typhimurium (strain LT2). Mol Microbiol 5:695–713. 1710759 10.1111/j.1365-2958.1991.tb00741.x

[33] Hardy GG, Toh E, Berne C, Brun YV 2018 Mutations in sugar-nucleotide synthesis genes restore holdfast polysaccharide anchoring to Caulobacter crescentus holdfast anchor mutants. J Bacteriol 200:e00597-17. doi:10.1128/JB.00597-17. 29158242 PMC5763047

[34] Walker SG, Karunaratne DN, Ravenscroft N, Smit J 1994 Characterization of mutants of Caulobacter crescentus defective in surface attachment of the paracrystalline surface layer. J Bacteriol 176:6312–6323. doi:10.1128/jb.176.20.6312-6323.1994. 7929003 PMC196973

[35] Dingwall A, Gober JW, Shapiro L 1990 Identification of a Caulobacter basal body structural gene and a cis-acting site required for activation of transcription. J Bacteriol 172:6066–6076. 2211524 10.1128/jb.172.10.6066-6076.1990 PMC526931

[36] Christen M, Beusch C, Bösch Y, Cerletti D, Flores-Tinoco CE, Del Medico L, Tschan F, Christen B 2016 Quantitative selection analysis of bacteriophage φCbK susceptibility in Caulobacter crescentus. J Mol Biol 428:419–430. doi:10.1016/j.jmb.2015.11.018. 26593064

[37] Haiko J, Westerlund-Wikström B 2013 The role of the bacterial flagellum in adhesion and virulence. Biology (Basel) 2:1242–1267. doi:10.3390/biology2041242. 24833223 PMC4009794

[38] Wang Y, Haitjema CH, Fuqua C 2014 The Ctp type IVb pilus locus of Agrobacterium tumefaciens directs formation of the common pili and contributes to reversible surface attachment. J Bacteriol 196:2979–2988. doi:10.1128/JB.01670-14. 24914181 PMC4135632

[39] Aldridge P, Paul R, Goymer P, Rainey P, Jenal U 2003 Role of the GGDEF regulator PleD in polar development of Caulobacter crescentus. Mol Microbiol 47:1695–1708. 12622822 10.1046/j.1365-2958.2003.03401.x

[40] Levi A, Jenal U 2006 Holdfast formation in motile swarmer cells optimizes surface attachment during Caulobacter crescentus development. J Bacteriol 188:5315–5318. doi:10.1128/JB.01725-05. 16816207 PMC1539976

[41] Berne C, Ellison CK, Agarwal R, Severin GB, Fiebig A, Morton RI III, Waters CM, Brun YV 6 8 2018 Feedback regulation of Caulobacter crescentus holdfast synthesis by flagellum assembly via the holdfast inhibitor HfiA. Mol Microbiol doi:10.1111/mmi.14099. PMC6195837 30079982

[42] Price MN, Wetmore KM, Waters RJ, Callaghan M, Ray J, Liu H, Kuehl JV, Melnyk RA, Lamson JS, Suh Y, Carlson HK, Esquivel Z, Sadeeshkumar H, Chakraborty R, Zane GM, Rubin BE, Wall JD, Visel A, Bristow J, Blow MJ, Arkin AP, Deutschbauer AM 2018 Mutant phenotypes for thousands of bacterial genes of unknown function. Nature 557:503–509. doi:10.1038/s41586-018-0124-0. 29769716

[43] Hoffman MD, Zucker LI, Brown PJB, Kysela DT, Brun YV, Jacobson SC 2015 Timescales and Frequencies of Reversible and Irreversible Adhesion Events of Single Bacterial Cells. Anal Chem 87:12032–12039. doi:10.1021/acs.analchem.5b02087. 26496389 PMC4756760

[44] Nesper J, Hug I, Kato S, Hee C-S, Habazettl JM, Manfredi P, Grzesiek S, Schirmer T, Emonet T, Jenal U 2017 Cyclic di-GMP differentially tunes a bacterial flagellar motor through a novel class of CheY-like regulators. Elife 6:e28842. doi:10.7554/eLife.28842. 29091032 PMC5677366

[45] Leclerc G, Wang SP, Ely B 1998 A new class of Caulobacter crescentus flagellar genes. J Bacteriol 180:5010–5019. 9748431 10.1128/jb.180.19.5010-5019.1998 PMC107534

[46] Kilmury SLN, Burrows LL 2016 Type IV pilins regulate their own expression via direct intramembrane interactions with the sensor kinase PilS. Proc Natl Acad Sci U S A 113:6017–6022. doi:10.1073/pnas.1512947113. 27162347 PMC4889343

[47] Aldridge P, Jenal U 1999 Cell cycle-dependent degradation of a flagellar motor component requires a novel-type response regulator. Mol Microbiol 32:379–391. 10231493 10.1046/j.1365-2958.1999.01358.x

[48] Paul R, Weiser S, Amiot NC, Chan C, Schirmer T, Giese B, Jenal U 2004 Cell cycle-dependent dynamic localization of a bacterial response regulator with a novel di-guanylate cyclase output domain. Genes Dev 18:715–727. doi:10.1101/gad.289504. 15075296 PMC387245

[49] Abel S, Bucher T, Nicollier M, Hug I, Kaever V, Abel Zur Wiesch P, Jenal U 2013 Bi-modal distribution of the second messenger c-di-GMP controls cell fate and asymmetry during the Caulobacter cell cycle. PLoS Genet 9:e1003744-17. doi:10.1371/journal.pgen.1003744. 24039597 PMC3764195

[50] O'Riordan K, Lee JC 2004 *Staphylococcus aureus* capsular polysaccharides. Clin Microbiol Rev 17:218–234. 14726462 10.1128/CMR.17.1.218-234.2004 PMC321462

[51] Stenutz R, Weintraub A, Widmalm G 2006 The structures of *Escherichia coli* O-polysaccharide antigens. FEMS Microbiol Rev 30:382–403. doi:10.1111/j.1574-6976.2006.00016.x. 16594963

[52] Olivier NB, Chen MM, Behr JR, Imperiali B 2006 In vitro biosynthesis of UDP- N,N′-diacetylbacillosamine by enzymes of the Campylobacter jejuni general protein glycosylation system. Biochemistry 45:13659–13669. doi:10.1021/bi061456h. 17087520 PMC2542654

[53] Mostafavi AZ, Troutman JM 2013 Biosynthetic assembly of the Bacteroides fragilis capsular polysaccharide A precursor bactoprenyl diphosphate-linked acetamido-4-amino-6-deoxygalactopyranose. Biochemistry 52:1939–1949. doi:10.1021/bi400126w. 23458065 PMC3629098

[54] Ely B 1991 Genetics of Caulobacter crescentus. Methods Enzymol 204:372–384. 1658564 10.1016/0076-6879(91)04019-k

[55] Gibson DG, Young L, Chuang R-Y, Venter JC, Hutchison CA, Smith HO 2009 Enzymatic assembly of DNA molecules up to several hundred kilobases. Nat Methods 6:343–345. doi:10.1038/nmeth.1318. 19363495

[56] Thanbichler M, Iniesta AA, Shapiro L 2007 A comprehensive set of plasmids for vanillate- and xylose-inducible gene expression in Caulobacter crescentus. Nucleic Acids Res 35:e137. doi:10.1093/nar/gkm818. 17959646 PMC2175322

[57] Hentchel KL, Ruiz LMR, Curtis PD, Fiebig A, Coleman ML, Crosson S 2019 Genome-scale fitness profile of Caulobacter crescentus grown in natural freshwater. ISME J 13:523–536. doi:10.1038/s41396-018-0295-6. 30297849 PMC6331620

[58] Eisen MB, Spellman PT, Brown PO, Botstein D 1998 Cluster analysis and display of genome-wide expression patterns. Proc Natl Acad Sci U S A 95:14863–14868. 9843981 10.1073/pnas.95.25.14863 PMC24541

[59] Walker SG, Smith SH, Smit J 1992 Isolation and comparison of the paracrystalline surface layer proteins of freshwater caulobacters. J Bacteriol 174:1783–1792. 1548228 10.1128/jb.174.6.1783-1792.1992 PMC205779

[60] Darveau RP, Hancock RE 1983 Procedure for isolation of bacterial lipopolysaccharides from both smooth and rough Pseudomonas aeruginosa and Salmonella typhimurium strains. J Bacteriol 155:831–838. 6409884 10.1128/jb.155.2.831-838.1983 PMC217756

[61] Kittelberger R, Hilbink F 1993 Sensitive silver-staining detection of bacterial lipopolysaccharides in polyacrylamide gels. J Biochem Biophys Methods 26:81–86. 8387076 10.1016/0165-022x(93)90024-i

[62] Foreman R, Fiebig A, Crosson S 2012 The LovK-LovR two-component system is a regulator of the general stress pathway in Caulobacter crescentus. J Bacteriol 194:3038–3049. doi:10.1128/JB.00182-12. 22408156 PMC3370868

