# Supplementary material for: A Genome-Wide Analysis of Adhesion in Caulobacter crescentus Identifies New Regulatory and Biosynthetic Components for Holdfast Assembly
Source: mBio. 2019 Feb 12;10(1):e02273-18. doi: 10.1128/mBio.02273-18 (PMC6372794; doi:10.1128/mBio.02273-18)
Supplement: TABLE S1 [file mBio.02273-18-st001.docx]

**Table S1** *Samples used for BarSeq analysis of gene fitness across cheesecloth passages*

The “Index” column represents the TruSeq indices used to de-multiplex the samples after sequencing. Cheese and PYE represent passages with and without cheesecloth, respectively. The numbers indicate which of the five passages each sample represents, and the letters indicate which of the three replicates. The “Proportion of reads in top percentile” column is calculated by ranking the barcodes in each sample by their abundance and determining the proportion of reads that map to the top 1% of barcodes.

| **Sample name** | **Index** | **Sample description** | **Total reads** | **Number of analyzable reads** | **Unique barcodes identified** | **Proportion of reads in top percentile** |
| --- | --- | --- | --- | --- | --- | --- |
| SCD_50.31 | CACGAT | PYE 1A | 4018890 | 3698106 | 233280 | 0.117 |
| SCD_50.32 | CACTCA | PYE 1B | 4492543 | 4130359 | 246118 | 0.1227 |
| SCD_50.33 | CAGGCG | PYE 1C | 4449306 | 4044087 | 150780 | 0.1236 |
| SCD_50.34 | CATGGC | PYE 2A | 4137845 | 3779486 | 226993 | 0.1249 |
| SCD_50.35 | CATTTT | PYE 2B | 4499135 | 4137664 | 228359 | 0.1274 |
| SCD_50.36 | CCAACA | PYE 2C | 4213003 | 3750243 | 228855 | 0.1316 |
| SCD_50.37 | CGGAAT | PYE 3A | 6735128 | 6171057 | 262618 | 0.1504 |
| SCD_50.38 | CTAGCT | PYE 3B | 6127591 | 5614129 | 249692 | 0.1471 |
| SCD_50.39 | CTATAC | PYE 3C | 5796738 | 5301288 | 246006 | 0.1563 |
| SCD_50.40 | CTCAGA | PYE 4A | 5322215 | 4857843 | 235579 | 0.1571 |
| SCD_50.41 | GACGAC | PYE 4B | 6394697 | 5855783 | 248801 | 0.1691 |
| SCD_50.42 | TAATCG | PYE 4C | 2975382 | 2755150 | 172812 | 0.1675 |
| SCD_50.43 | TACAGC | PYE 5A | 5799597 | 5301223 | 246151 | 0.2002 |
| SCD_50.44 | TATAAT | PYE 5B | 6016430 | 5569218 | 234947 | 0.1972 |
| SCD_50.45 | TCATTC | PYE 5C | 4811202 | 4428232 | 209890 | 0.2178 |
| SCD_50.46 | TCCCGA | Cheese 1A | 4562846 | 4108435 | 242916 | 0.1455 |
| SCD_50.47 | TCGAAG | Cheese 1B | 4313848 | 3922295 | 233599 | 0.1484 |
| SCD_50.48 | TCGGCA | Cheese 1C | 4773752 | 4335186 | 247032 | 0.1516 |
| SCD_50.49 | AAACAT | Cheese 2A | 6050903 | 5557666 | 232487 | 0.2488 |
| SCD_50.50 | AAAGCA | Cheese 2B | 7013967 | 6384659 | 253431 | 0.2793 |
| SCD_50.51 | AAATGC | Cheese 2C | 4421479 | 4054218 | 211068 | 0.2713 |
| SCD_50.52 | AACAAA | Cheese 3A | 6616953 | 5904400 | 235379 | 0.4118 |
| SCD_50.53 | AACTTG | Cheese 3B | 5935535 | 5450038 | 209707 | 0.4585 |
| SCD_50.54 | AAGACT | Cheese 3C | 7750956 | 7158882 | 204209 | 0.5887 |
| SCD_50.55 | AAGCGA | Cheese 4A | 4002747 | 3664336 | 167909 | 0.6349 |
| SCD_50.56 | AAGGAC | Cheese 4B | 3156990 | 2902069 | 151371 | 0.605 |
| SCD_50.57 | AATAGG | Cheese 4C | 1882499 | 1730719 | 115437 | 0.6777 |
| SCD_50.58 | ACAAAC | Cheese 5A | 2475323 | 2197424 | 99108 | 0.787 |
| SCD_50.59 | ACATCT | Cheese 5B | 2419554 | 2241238 | 99833 | 0.7796 |
| SCD_5060 | ACCCAG | Cheese 5C | 2865211 | 2583906 | 107021 | 0.8014 |
