# Supplementary material for: A Genome-Wide Analysis of Adhesion in Caulobacter crescentus Identifies New Regulatory and Biosynthetic Components for Holdfast Assembly
Source: mBio. 2019 Feb 12;10(1):e02273-18. doi: 10.1128/mBio.02273-18 (PMC6372794; doi:10.1128/mBio.02273-18)
Supplement: TABLE S5 [file mBio.02273-18-st005.docx]

**Table S5** *Fitness scores across cheesecloth passages for mutant clusters shown in Fig 1C*

Fitness values represent the average of the three replicates for each passage. The first passage without cheesecloth (PYE1) represents the time 0 sample.

| ***Cluster 1: SLPS*** |  |  |  |  |  |  |
| --- | --- | --- | --- | --- | --- | --- |
| **locusId; annotation** | **0** | **1** | **2** | **3** | **4** | **5** |
| CCNA_00044; ribosome maturation protein RimP | 0.013 | -2.93 | -4.145 | -3.319 | -1.57 | -0.067 |
| CCNA_00217; thiol:disulfide interchange protein DsbD | -0.07 | -2.488 | -2.869 | -1.883 | -0.313 | 1.215 |
| CCNA_00290; autotransporter protein | 0.009 | -3.141 | -3.74 | -3.294 | -2.788 | -1.794 |
| CCNA_00390; ADP-heptose--LPS heptosyltransferase | -0.008 | -2.321 | -4.402 | -2.913 | -2.413 | -1.408 |
| CCNA_00497; putative rhamnosyl transferase | -0.074 | -3.219 | -3.706 | -2.835 | -1.19 | 0.371 |
| CCNA_00502; glycosyl transferase family protein | 0.024 | -3.405 | -3.06 | -3.883 | -2.296 | -1.313 |
| CCNA_00512; GTP-binding protein, probable translation factor | 0.082 | -3.203 | -3.936 | -3.087 | -1.455 | 0.104 |
| CCNA_00519; conserved hypothetical protein | 0.009 | -4.406 | -4.912 | -4.798 | -3.134 | -1.521 |
| CCNA_00667; lipopolysaccharide biosynthesis protein | -0.194 | -2.497 | -2.358 | -1.846 | -0.306 | 1.286 |
| CCNA_00668; capsular polysaccharide biosynthesis protein | -0.001 | -2.031 | -3.171 | -3.547 | -1.836 | -1.285 |
| CCNA_00669; glycosyltransferase family 99 protein WbsX | -0.022 | -3.396 | -3.205 | -2.343 | -0.788 | 0.792 |
| CCNA_01055; GT1 family glyscosyl transferase | -0.043 | -3.088 | -4.106 | -3.578 | -2.06 | -0.533 |
| CCNA_01062; GDP-mannose 4,6 dehydratase | -0.117 | -3.685 | -4.072 | -3.613 | -1.995 | -0.595 |
| CCNA_01063; UDP-perosamine 4-acetyl transferase | -0.038 | -3.41 | -2.969 | -2.665 | -0.986 | 0.2 |
| CCNA_01064; perosamine synthetase | -0.112 | -3.481 | -4 | -3.214 | -1.594 | -0.064 |
| CCNA_01065; glycosyltransferase | -0.076 | -3.761 | -3.547 | -3.178 | -1.581 | -0.043 |
| CCNA_01066; glycosyltransferase | -0.038 | -2.802 | -3.277 | -2.57 | -1.632 | -0.394 |
| CCNA_01068; glycosyltransferase | -0.15 | -4.496 | -3.787 | -3.662 | -1.324 | -0.508 |
| CCNA_01086; GTP-binding protein lepA | 0.043 | -3.449 | -3.931 | -3.509 | -2.112 | -0.57 |
| CCNA_01103; ADP-heptose--LPS heptosyltransferase | 0.034 | -2.691 | -2.823 | -3.069 | -1.778 | -0.077 |
| CCNA_01199; glucose-1-phosphate thymidylyltransferase | -0.008 | -4.037 | -3.851 | -3.099 | -1.424 | 0.062 |
| CCNA_01375; lactoylglutathione lyase | 0.048 | -3.392 | -4.654 | -4.066 | -2.463 | -1.058 |
| CCNA_01427; beta-barrel assembly machine (BAM) protein BamE | 0.052 | -2.633 | -4.028 | -4.39 | -2.727 | -1.235 |
| CCNA_01430; conserved hypothetical protein | -0.013 | -1.923 | -2.959 | -3.144 | -2.08 | -0.903 |
| CCNA_01447; homoserine dehydrogenase | 0.02 | -2.348 | -3.724 | -2.91 | -1.286 | 0.261 |
| CCNA_01497; ADP-L-glycero-D-manno-heptose-6-epimerase | 0.022 | -2.19 | -2.593 | -2.199 | -1.98 | -0.42 |
| CCNA_01955; zinc metalloprotease | 0.005 | -2.151 | -2.316 | -2.232 | -1.865 | -1.314 |
| CCNA_01971; peptidyl-prolyl cis-trans isomerase | 0.013 | -2.17 | -2.493 | -1.791 | -0.927 | 0.591 |
| CCNA_02219; hypothetical protein | 0.017 | -2.85 | -2.949 | -2.521 | -2.268 | -1.687 |
| CCNA_02326; acetylornithine aminotransferase/succinyldiaminopimelate aminotransferase | 0.019 | -1.712 | -2.166 | -1.194 | -0.525 | 0.791 |
| CCNA_02347; phosphomannomutase/phosphoglucomutase | -0.064 | -4.09 | -4.85 | -4.033 | -2.455 | -0.889 |
| CCNA_02386; O-antigen ligase related enzyme | -0.134 | -2.762 | -3.457 | -2.585 | -1.025 | 0.537 |
| CCNA_02463; UDP-N-acetylglucosamine 4-epimerase | 0.029 | -2.627 | -3.781 | -3.758 | -2.374 | -0.882 |
| CCNA_02650; N-acetyl-anhydromuramyl-L-alanine amidase | -0.018 | -1.617 | -3.035 | -2.194 | -0.678 | 0.911 |
| CCNA_02941; transcription elongation factor greA | -0.119 | -1.233 | -2.748 | -2.498 | -0.92 | 0.629 |
| CCNA_03026; two-component response regulator petR | -0.055 | -2.619 | -4.063 | -3.652 | -2.577 | -1.705 |
| CCNA_03195; RNA polymerase sigma factor RpoH | -0.001 | -2.089 | -3.735 | -3.307 | -1.951 | -1.294 |
| CCNA_03352; YebC/PmpR transcriptional regulator | -0.009 | -3.942 | -4.169 | -3.705 | -2.805 | -1.706 |
| CCNA_03475; homoserine kinase | -0.028 | -1.569 | -3.168 | -2.323 | -0.697 | 0.84 |
| CCNA_03609; outer membrane protein | -0.011 | -2.28 | -3.936 | -3.203 | -1.489 | -0.261 |
| CCNA_03705; conserved hypothetical protein | 0.053 | -1.304 | -3.383 | -1.826 | -0.864 | 0.665 |
| CCNA_03713; RNA polymerase sigma-54 factor rpoN | 0.02 | -1.566 | -3.418 | -2.288 | -1.299 | 0.198 |
| CCNA_03733; mannose-1-phosphate guanylyltransferase | -0.137 | -2.754 | -3.111 | -2.202 | -1.085 | 0.29 |
| CCNA_03744; dTDP-glucose 4,6-dehydratase | -0.009 | -2.964 | -3.124 | -1.88 | -1.002 | 0.388 |
| CCNA_03748; dTDP-4-dehydrorhamnose 3,5-epimerase | -0.059 | -2.155 | -2.242 | -1.967 | -0.249 | 0.731 |
| CCNA_03859; two-component response regulator cenR | 0.01 | -1.898 | -3.244 | -2.126 | -0.879 | 0.579 |
| CCNA_03909; conserved hypothetical protein | 0.028 | -2.113 | -4.213 | -2.917 | -1.893 | -0.234 |
| CCNA_03984; hypothetical protein | 0.007 | -1.779 | -2.747 | -2.011 | -0.292 | -0.697 |
|  |  |  |  |  |  |  |
| ***Cluster 2: Polar appendages*** |  |  |  |  |  |  |
| **locusId; annotation** | **0** | **1** | **2** | **3** | **4** | **5** |
| CCNA_00233; UDP-N-acetylglucosamine 4,6-dehydratase | 0.008 | -0.398 | -0.647 | -1.464 | -1.536 | -1.994 |
| CCNA_00234; WecE-family cell wall biogenesis enzyme | 0.005 | -0.587 | -1.119 | -1.518 | -1.539 | -2.71 |
| CCNA_00444; chemotaxis protein methyltransferase | -0.017 | -0.73 | -1.261 | -1.337 | -1.85 | -2.405 |
| CCNA_00447; chemotaxis protein cheD | -0.008 | -0.393 | -1.262 | -1.868 | -2.689 | -2.926 |
| CCNA_00449; cheYIII | -0.001 | -0.649 | -1.243 | -1.71 | -2.675 | -2.985 |
| CCNA_00542; hypothetical protein | 0.003 | -0.471 | -1.169 | -1.452 | -1.965 | -2.529 |
| CCNA_00787; chemotaxis motA protein | -0.012 | -0.721 | -0.806 | -1.894 | -2.588 | -3.396 |
| CCNA_00821; hypothetical protein | -0.027 | -0.613 | -1.472 | -0.837 | -1.245 | -2.046 |
| CCNA_00823; LuxR-like DNA-binding protein | -0.058 | -0.97 | -1.218 | -1.518 | -2.066 | -2.612 |
| CCNA_00942; flagellar hook-associated protein FlgL | 0.004 | -0.424 | -0.765 | -1.385 | -1.835 | -2.326 |
| CCNA_00943; flagellar hook-associated protein FlaN | -0.032 | 0.105 | -0.548 | -0.928 | -2.707 | -2.553 |
| CCNA_01004; flagellar basal-body rod protein FlgB | 0.004 | -0.37 | -0.935 | -1.357 | -2.617 | -3.031 |
| CCNA_01005; flagellar basal-body rod protein flgC | 0.004 | -0.426 | -0.738 | -1.599 | -2.121 | -3.296 |
| CCNA_01094; hypothetical protein | -0.002 | 0.146 | -0.421 | -1.277 | -1.473 | -3.97 |
| CCNA_01117; conserved hypothetical protein | 0.006 | -0.472 | -0.715 | -1.256 | -1.147 | -1.777 |
| CCNA_01524; FlbA protein | 0.001 | -0.427 | -0.921 | -1.919 | -1.998 | -2.886 |
| CCNA_01527; flagellin fljL | 0.011 | -0.601 | -1.477 | -1.912 | -2.791 | -2.989 |
| CCNA_01530; flagellin FljJ | -0.005 | -0.245 | -0.754 | -1.332 | -1.409 | -2.203 |
| CCNA_01532; regulatory protein flaY | 0.004 | -0.56 | -0.887 | -1.34 | -1.497 | -1.985 |
| CCNA_01562; 4-hydroxy-2-oxoglutarate aldolase/2-dehydro-3-deoxyphosphogluconate aldolase | -0.001 | 0.111 | -0.489 | -1.482 | -2.752 | -3.307 |
| CCNA_01644; chemotaxis motB protein | -0.002 | -0.479 | -0.629 | -1.493 | -2.599 | -2.458 |
| CCNA_01675; outer membrane protein | -0.008 | -0.328 | -0.695 | -1.409 | -1.926 | -2.472 |
| CCNA_01676; conserved hypothetical protein | 0.006 | -0.455 | -0.55 | -1.207 | -2.14 | -2.362 |
| CCNA_02142; flagellar basal-body rod protein flgF | -0.011 | -0.368 | -1.402 | -1.304 | -2.054 | -3.858 |
| CCNA_02143; flagellar basal-body rod protein flgG | -0.008 | -0.273 | -1.237 | -1.728 | -2.163 | -2.703 |
| CCNA_02144; flagella basal body P ring formation protein flgA | 0.004 | -0.357 | -0.887 | -1.701 | -1.911 | -3.179 |
| CCNA_02145; flagellar L-ring protein flgH | -0.005 | -0.473 | -1.193 | -1.891 | -2.175 | -3.406 |
| CCNA_02322; Co2+/Mg2+ efflux protein ApaG | 0.01 | -0.146 | -0.529 | -0.943 | -1.572 | -2.072 |
| CCNA_02411; putative lytic transglycosylase PleA | 0.008 | -0.039 | -0.349 | -0.84 | -1.079 | -1.064 |
| CCNA_02526; dihydroorotase | 0 | -0.85 | -0.636 | -1.913 | -2.573 | -3.177 |
| CCNA_02667; flagellar basal-body protein FlbY | -0.011 | -0.328 | -0.781 | -1.413 | -1.898 | -3.416 |
| CCNA_02796; conserved hypothetical protein | -0.019 | -0.623 | -1.316 | -2.031 | -2.836 | -4.51 |
| CCNA_02946; spsF-related cytidylyltransferase | 0.002 | -0.342 | -0.765 | -1.462 | -1.667 | -1.885 |
| CCNA_02947; spsG-related polysaccharide biosynthesis protein | 0.007 | -0.126 | -0.843 | -0.56 | -2.42 | -2.072 |
| CCNA_02950; hypothetical protein | 0.006 | -0.564 | -1.334 | -1.874 | -2.604 | -2.534 |
| CCNA_02951; WbqC-like family protein | 0.011 | -0.302 | -0.744 | -1.461 | -1.905 | -2.271 |
| CCNA_02961; NeuB-family N-acetylneuraminate synthase | 0.005 | -0.404 | -0.841 | -1.145 | -2.067 | -2.738 |
| CCNA_03035; TadC-related pilus assembly protein | 0.013 | -0.501 | -0.716 | -1.382 | -1.531 | -1.851 |
| CCNA_03036; TadB-related pilus assembly protein | 0.004 | -0.411 | -0.608 | -1.411 | -1.647 | -2.348 |
| CCNA_03041; pilus assembly protein CpaB | 0.004 | -0.193 | -0.661 | -0.824 | -0.946 | -1.653 |
| CCNA_03890; conserved hypothetical protein | 0.007 | 0.049 | -0.268 | -1.598 | -1.602 | -2.476 |
|  |  |  |  |  |  |  |
| ***Cluster 3: Pilus assembly*** |  |  |  |  |  |  |
| **locusId; annotation** | **0** | **1** | **2** | **3** | **4** | **5** |
| CCNA_03033; Flp pilus assembly protein TadD | 0.008 | 0.009 | -0.62 | -0.629 | -0.758 | -1.594 |
| CCNA_03035; TadC-related pilus assembly protein | 0.013 | -0.501 | -0.716 | -1.382 | -1.531 | -1.851 |
| CCNA_03036; TadB-related pilus assembly protein | 0.004 | -0.411 | -0.608 | -1.411 | -1.647 | -2.348 |
| CCNA_03037; pilus assembly ATPase CpaF | 0.005 | 0.095 | -0.253 | -0.32 | -0.409 | -0.403 |
| CCNA_03038; pilus assembly ATPase CpaE | -0.004 | -0.237 | -0.211 | -0.606 | -0.457 | -1.147 |
| CCNA_03039; pilus assembly protein CpaD | 0.037 | -0.125 | -0.317 | -0.922 | -1.06 | -0.943 |
| CCNA_03040; outer membrane pilus secretion channel CpaC | 0.008 | -0.244 | -0.478 | -0.827 | -1.23 | -1.472 |
| CCNA_03041; pilus assembly protein CpaB | 0.004 | -0.193 | -0.661 | -0.824 | -0.946 | -1.653 |
| CCNA_03042; pilus assembly prepilin peptidase CpaA | -0.001 | -0.267 | 0.188 | 0.554 | 0.736 | 0.911 |
| CCNA_03043; type IV pilin protein pilA | 0.006 | 0.615 | 1.513 | 2.023 | 2.366 | 2.569 |
| CCNA_03044; CpaC-related secretion pathway protein | 0.008 | -0.301 | -0.433 | -1.517 | -1.481 | -1.527 |
| CCNA_03045; TadG-related pilus assembly protein | 0 | 0.045 | -0.047 | 0.016 | -0.154 | 0.109 |
| CCNA_03046; TadE-related pilus assembly protein | -0.004 | -0.33 | -0.304 | -0.389 | -0.182 | 0.06 |
|  |  |  |  |  |  |  |
| ***Cluster 4: Holdfast synthesis*** |  |  |  |  |  |  |
| **locusId; annotation** | **0** | **1** | **2** | **3** | **4** | **5** |
| CCNA_00094; WecG/TagA-family glycosyltransferase HfsJ | -0.001 | 1.792 | 3.826 | 5.461 | 6.707 | 7.85 |
| CCNA_01241; Zn-dependent hydrolase, glyoxalase II family | -0.014 | 0.565 | 2.753 | 4.296 | 5.334 | 6.595 |
| CCNA_01242; amino acid permease | 0.008 | 2.034 | 5.865 | 8.127 | 8.789 | 9.649 |
| CCNA_02360; glycosyl transferase family 2 protein | 0.006 | 1.507 | 2.857 | 3.987 | 4.727 | 5.919 |
| CCNA_02436; hypothetical protein | -0.011 | 0.37 | 1.598 | 2.491 | 4.207 | 5.78 |
| CCNA_02509; glycosyltransferase hfsG | 0 | 1.44 | 2.961 | 4.334 | 5.264 | 6.452 |
| CCNA_02510; oligosaccharide deacetylase hfsH | -0.002 | 1.328 | 3.155 | 4.619 | 5.782 | 6.993 |
| CCNA_02512; polysaccharide autokinase-related protein hfsB | -0.005 | 1.819 | 3.721 | 5.31 | 6.57 | 7.798 |
| CCNA_02513; holdfast synthesis protein HfsA | -0.002 | 1.823 | 3.702 | 5.359 | 6.375 | 7.653 |
| CCNA_02514; polysaccharide secretin protein hfsD | 0.001 | 1.8 | 3.812 | 5.491 | 6.638 | 7.911 |
| CCNA_02567; sensory transduction histidine kinase pleC | 0.002 | 2.23 | 4.093 | 5.298 | 6.293 | 7.414 |
| CCNA_02711; holdfast attachment protein hfaA | -0.019 | 1.335 | 2.869 | 4.649 | 5.335 | 6.242 |
| CCNA_02712; holdfast attachment protein hfaB | 0.001 | 1.441 | 3.288 | 4.825 | 5.894 | 7.119 |
| CCNA_02713; holdfast attachment protein hfaD | 0.004 | 1.053 | 2.76 | 3.972 | 5.004 | 5.894 |
|  |  |  |  |  |  |  |
| ***Cluster 5: Holdfast modification*** |  |  |  |  |  |  |
| **locusId; annotation** | **0** | **1** | **2** | **3** | **4** | **5** |
| CCNA_00006; enoyl-CoA hydratase | 0.006 | 1.416 | 2.106 | 2.633 | 2.838 | 2.885 |
| CCNA_00011; chaperone protein DnaJ | -0.002 | 0.391 | 1.424 | 2.487 | 3.42 | 3.55 |
| CCNA_00134; surface protein | 0.012 | 1.004 | 1.223 | 2.175 | 2.703 | 3.104 |
| CCNA_00135; trypsin-like peptidase | 0.024 | 1.408 | 1.849 | 2.536 | 2.983 | 3.508 |
| CCNA_00247; two-component receiver protein SpdR | -0.022 | 0.746 | 1.113 | 2.001 | 1.936 | 2.122 |
| CCNA_00527; conserved hypothetical protein | 0.015 | 0.266 | 0.896 | 2.136 | 3.469 | 4.796 |
| CCNA_00543; methyl-accepting chemotaxis protein | 0.009 | 1.134 | 1.716 | 2.128 | 2.157 | 2.226 |
| CCNA_00551; hypothetical protein | -0.004 | 0.866 | 1.543 | 2.139 | 2.308 | 2.69 |
| CCNA_00554; methyltransferase | -0.001 | 0.062 | 0.822 | 2.006 | 2.545 | 2.978 |
| CCNA_00908; 3-oxoacyl-(acyl-carrier-protein) synthase III | 0 | 0.407 | 0.586 | 0.845 | 1.489 | 2.322 |
| CCNA_00948; CtrA inhibitory protein SciP | 0.007 | 1.162 | 1.949 | 2.067 | 2.008 | 2.039 |
| CCNA_01020; LacI-family transcriptional regulator | 0.007 | 0.551 | 1.386 | 2.301 | 2.185 | 2.402 |
| CCNA_01214; YjgP/YjgQ family membrane permease | 0.015 | 2.881 | 3.481 | 3.156 | 3.126 | 3.309 |
| CCNA_01215; histidine triad (HIT) hydrolase | 0.028 | 1.556 | 1.748 | 1.705 | 2.032 | 2.724 |
| CCNA_01345; short chain dehydrogenase | 0.005 | 0.092 | 0.3 | 0.598 | 1.441 | 2.418 |
| CCNA_01354; myo-inositol 2-dehydrogenase IdhA | 0.011 | 0.138 | 0.551 | 0.717 | 1.425 | 2.056 |
| CCNA_01893; SnoaL-like domain protein | -0.014 | 0.189 | 0.498 | 0.484 | 1.23 | 2.187 |
| CCNA_02087; deoxyguanosinetriphosphate triphosphohydrolase | 0.002 | 0.592 | 1.448 | 1.871 | 2.344 | 2.614 |
| CCNA_02125; polar development protein podJ | 0.008 | 1.149 | 2.18 | 2.796 | 3.456 | 4.419 |
| CCNA_02242; PHB granule-associated protein, phasin2 | -0.018 | 0.695 | 1.362 | 1.519 | 1.819 | 2.214 |
| CCNA_02415; Xre-family transcriptional regulator | 0.001 | -0.156 | 2.237 | 3.038 | 2.641 | 2.593 |
| CCNA_02619; stomatin/prohibitin-related protein | -0.013 | 0.45 | 1.896 | 3.271 | 3.38 | 3.866 |
| CCNA_02722; conserved hypothetical protein | 0.002 | 0.867 | 1.936 | 2.676 | 3.336 | 4.092 |
| CCNA_02846; DegP/HtrA-family serine protease | 0.018 | 0.267 | 1.041 | 1.554 | 1.965 | 2.587 |
| CCNA_02880; terminase-like family protein | -0.003 | 0.224 | 0.389 | 0.732 | 1.32 | 2.122 |
| CCNA_02934; conserved hypothetical protein | -0.003 | 0.633 | 1.144 | 2.146 | 2.127 | 2.572 |
| CCNA_03099; hypothetical protein | 0.002 | 0.378 | 0.678 | 1.271 | 1.525 | 2.473 |
| CCNA_03161; transcriptional regulator xylR | 0.017 | 1.201 | 1.769 | 2.792 | 2.9 | 2.983 |
| CCNA_03386; multimodular transpeptidase-transglycosylase PbpC | 0.002 | 0.775 | 1.546 | 2.13 | 2.477 | 2.602 |
| CCNA_03465; glycine cleavage system aminomethyltransferase T | 0.012 | 1.186 | 1.447 | 1.937 | 2.076 | 2.344 |
| CCNA_03611; glutathione-regulated potassium-efflux system protein kefC | 0.005 | -0.076 | 1.577 | 2.82 | 3.551 | 3.907 |
| CCNA_03803; acetyltransferase family holdfast biogenesis protein HfsK | -0.003 | 0.895 | 1.957 | 2.71 | 3.132 | 3.698 |
| CCNA_03902; conserved hypothetical protein | -0.009 | 0.681 | 1.566 | 2.037 | 2.339 | 3.165 |
