# Supplementary figures and images for: A Genome-Wide Analysis of Adhesion in Caulobacter crescentus Identifies New Regulatory and Biosynthetic Components for Holdfast Assembly
Source: mBio. 2019 Feb 12;10(1):e02273-18. doi: 10.1128/mBio.02273-18 (PMC6372794; doi:10.1128/mBio.02273-18)

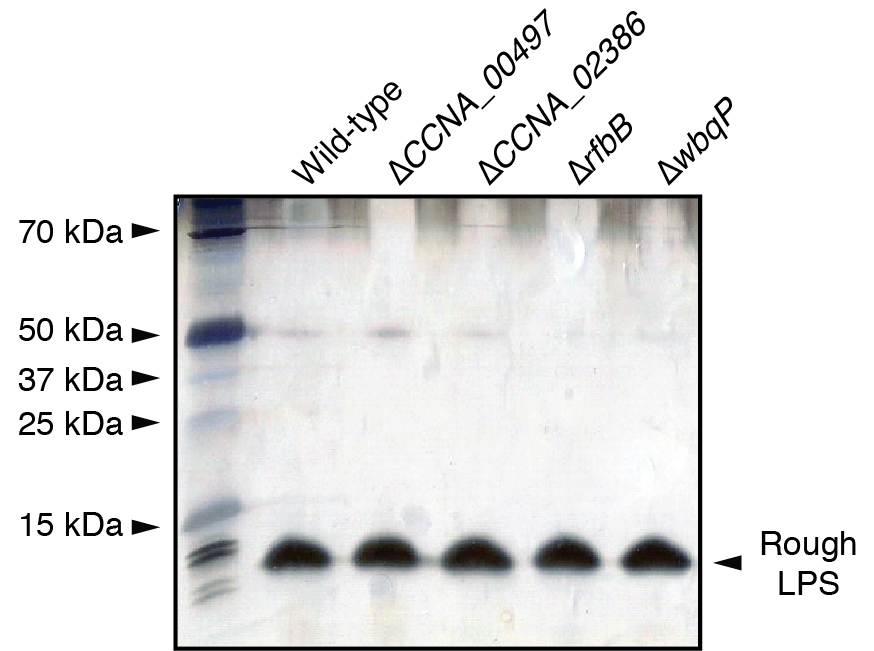

Supplement: FIG S1 [file mBio.02273-18-sf001.tif]

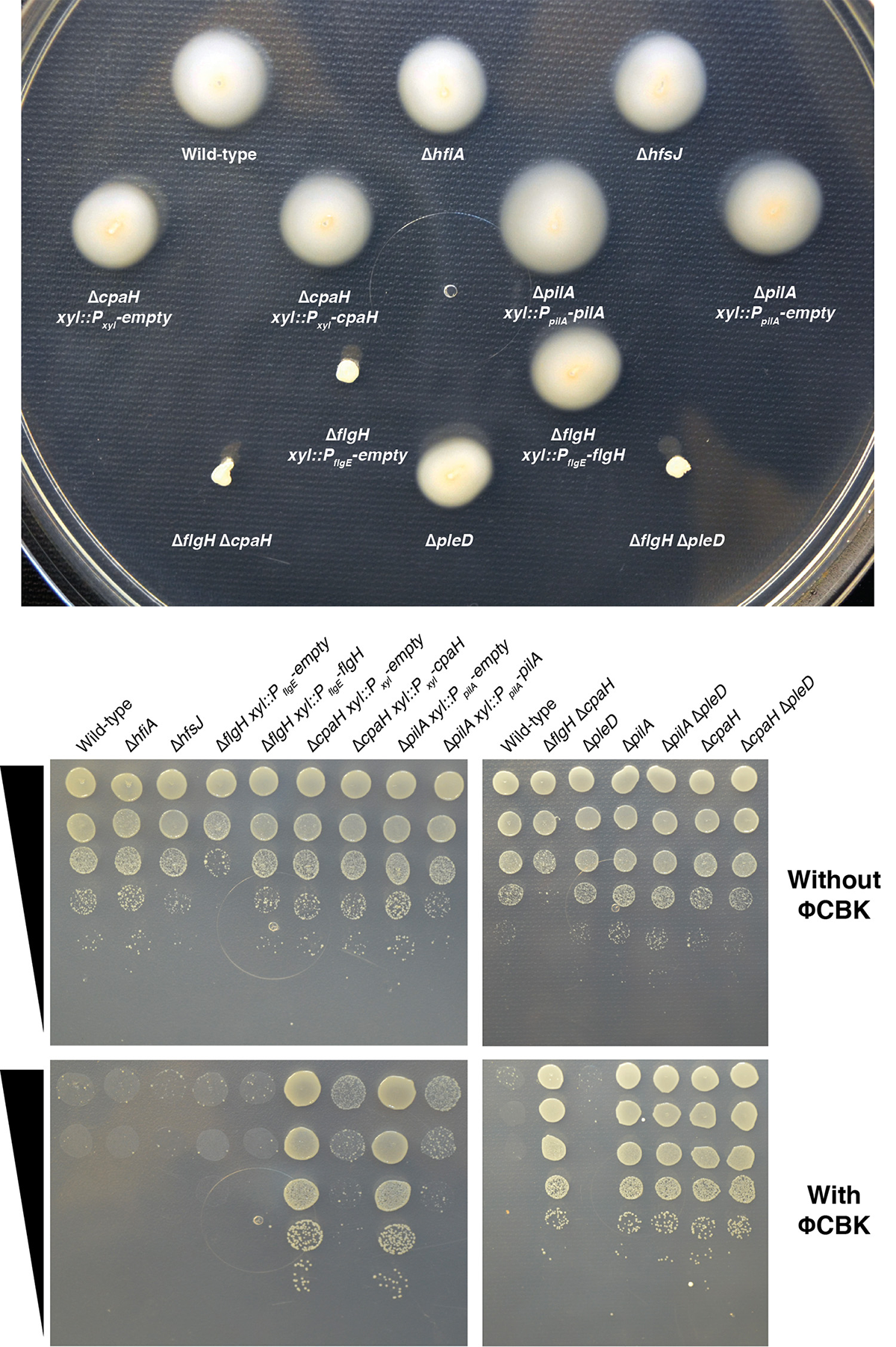

Supplement: FIG S2 [file mBio.02273-18-sf002.tif]

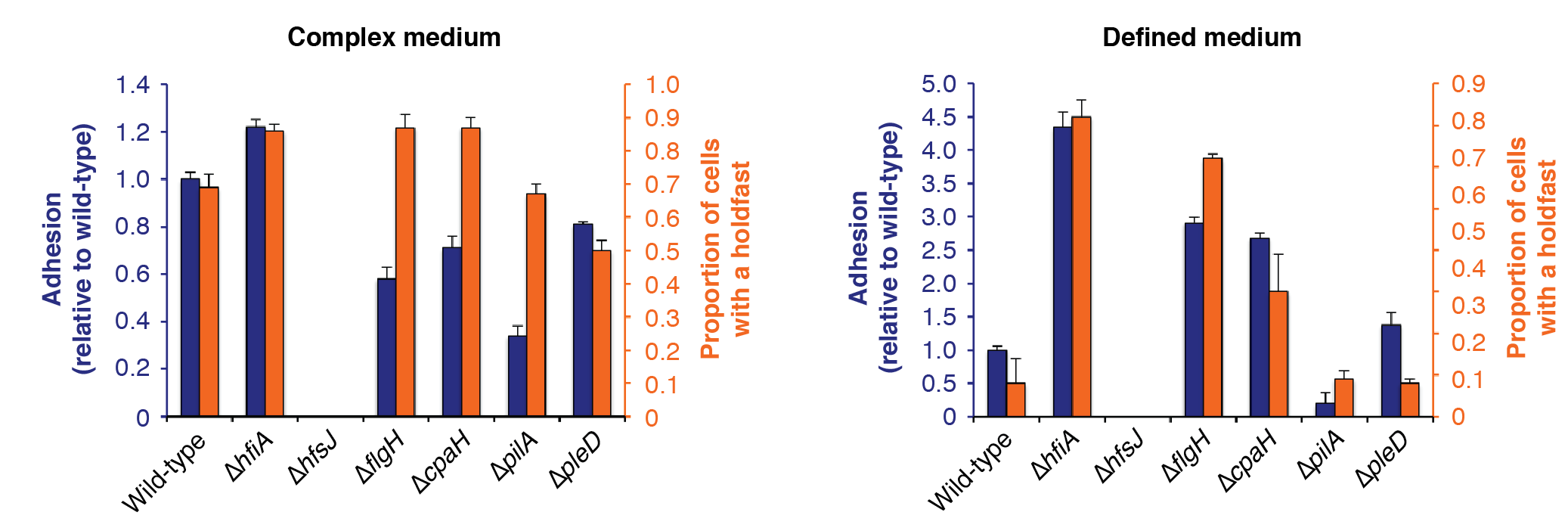

Supplement: FIG S3 [file mBio.02273-18-sf003.tif]

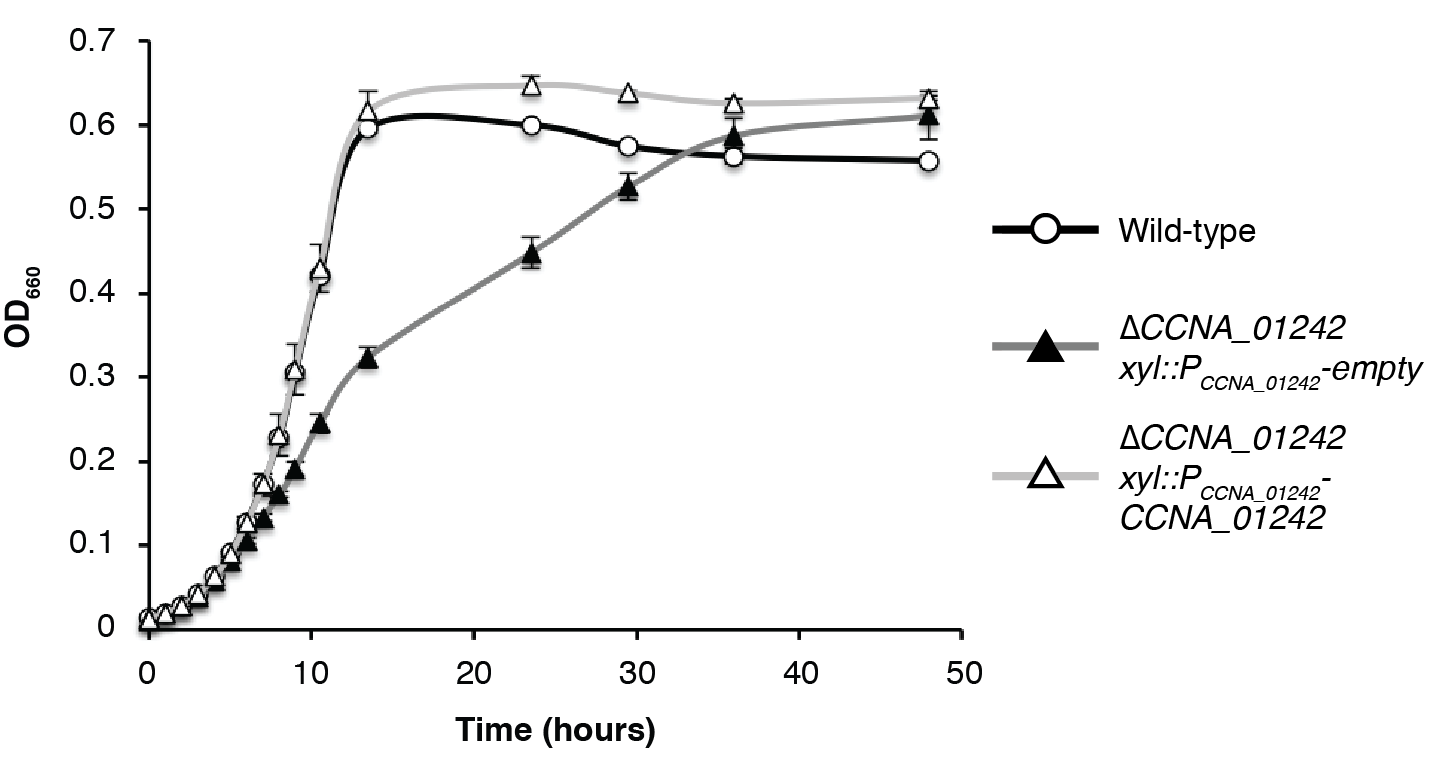

Supplement: FIG S4 [file mBio.02273-18-sf004.tif]
