# Supplementary material for: A Genome-Wide Analysis of Adhesion in Caulobacter crescentus Identifies New Regulatory and Biosynthetic Components for Holdfast Assembly
Source: mBio. 2019 Feb 12;10(1):e02273-18. doi: 10.1128/mBio.02273-18 (PMC6372794; doi:10.1128/mBio.02273-18)
Supplement: TABLE S3 [file mBio.02273-18-st003.docx]

**Table S3** *Holdfast counts for polar appendage mutants­­*

The values represent the fraction of cells (out of 1) that stained with a holdfast focus along with the associated standard deviation from three biological replicates. The total number of cells counted is shown in parentheses. Cells were harvested from low-density cultures (Materials and Methods) to minimize the formation of rosettes. However, in the event that rosettes were observed, all cells in the rosette were counted as holdfast producing.

| **Strain** | **PYE** | **M2X** |
| --- | --- | --- |
| Wild-type | 0.686 ± 0.045 (739) | 0.081 ± 0.006 (743) |
| ∆*hfiA* | 0.863 ± 0.021 (526) | 0.722 ± 0.039 (703) |
| ∆*hfsJ* | 0.000 ± 0.000 (628) | 0.000 ± 0.000 (638) |
| ∆*flgH* | 0.869 ± 0.037 (662) | 0.620 ± 0.013 (609) |
| ∆*cpaH* | 0.874 ± 0.031 (531) | 0.296 ± 0.094 (689) |
| ∆*pilA* | 0.673 ± 0.030 (464) | 0.092 ± 0.016 (367) |
| ∆*pleD* | 0.496 ± 0.024 (457) | 0.084 ± 0.007 (500) |
