# Supplementary material for: A Genome-Wide Analysis of Adhesion in Caulobacter crescentus Identifies New Regulatory and Biosynthetic Components for Holdfast Assembly
Source: mBio. 2019 Feb 12;10(1):e02273-18. doi: 10.1128/mBio.02273-18 (PMC6372794; doi:10.1128/mBio.02273-18)
Supplement: TABLE S4 [file mBio.02273-18-st004.docx]

**Table S4** *Additional adhesion phenotypes for polar appendage mutants*

Normalized crystal violet staining values are shown as the average ± standard deviation from at least 4 biological replicates. All values shown reflect trends that were consistent across at least five independent experiments. Cells were grown for 17 hours in M2X or 24 hours in PYE medium before staining. n.m. – not measured.

| **Genotype** | **Deletion (PYE)** | **∆hfiA (PYE)** | **∆pleD (PYE)** | **Deletion (M2X)** | **∆hfiA (M2X)** | **∆pleD (M2X)** |
| --- | --- | --- | --- | --- | --- | --- |
| Wild-type | 1.00 ± 0.03 | 1.28 ± 0.16 | 0.81 ± 0.01 | 1.00 ± 0.06 | 5.61 ± 0.27 | 1.38 ± 0.19 |
| ∆*hfiA* | 1.28 ± 0.16 | n.m. | 1.08 ± 0.07 | 4.34 ± 0.23 | n.m. | 5.53 ± 0.16 |
| ∆*hfsJ* | 0.00 ± 0.00 | n.m. | n.m. | 0.00 ± 0.00 | n.m. | n.m. |
| ∆*wbqP* ∆*hfsJ* | 0.48 ± 0.25 | n.m. | n.m. | 2.51 ± 0.64 | n.m. | n.m. |
| ∆*flgH* | 0.58 ± 0.05 | 0.62 ± 0.07 | 0.31 ± 0.02 | 2.91 ± 0.08 | 2.78 ± 0.21 | 1.41 ± 0.33 |
| ∆*cpaH* | 0.71 ± 0.05 | 0.91 ± 0.05 | 0.19 ± 0.01 | 2.68 ± 0.07 | 4.29 ± 0.16 | 1.22 ± 0.18 |
| ∆*pilA* | 0.34 ± 0.04 | 0.73 ± 0.03 | 0.43 ± 0.01 | 0.20 ± 0.16 | 5.95 ± 0.18 | 0.25 ± 0.08 |
| ∆*flgH* ∆*hfsJ* | 0.00 ± 0.00 | n.m. | n.m. | 0.01 ± 0.01 | n.m. | n.m. |
| ∆*cpaH* ∆*hfsJ* | 0.00 ± 0.00 | n.m. | n.m. | 0.01 ± 0.02 | n.m. | n.m. |
| ∆*flgH* ∆*cpaH* | 0.40 ± 0.01 | n.m. | n.m. | 4.13 ± 0.18 | n.m. | n.m. |
| ∆*flgH* ∆*pilA* | 0.32 ± 0.02 | n.m. | n.m. | 2.00 ± 0.25 | n.m. | n.m. |
| ∆*cpaH* ∆*pilA* | 0.43 ± 0.03 | n.m. | n.m. | 0.12 ± 0.04 | n.m. | n.m. |
