# Supplementary material for: A Genome-Wide Analysis of Adhesion in Caulobacter crescentus Identifies New Regulatory and Biosynthetic Components for Holdfast Assembly
Source: mBio. 2019 Feb 12;10(1):e02273-18. doi: 10.1128/mBio.02273-18 (PMC6372794; doi:10.1128/mBio.02273-18)
Supplement: TABLE S6 [file mBio.02273-18-st006.docx]

**Table S6** *Strains and plasmids used in this study*

To analyze the sequence data used for fitness calculations, we used the *C. crescentus* NA1000 genome annotations. NA1000 is directly derived from CB15, but its genome has more detailed annotations and is better curated. To facilitate interpretation of the BarSeq data, genotypes in the text and supplemental tables use the NA1000 locus numbers and nomenclature. We note the corresponding CB15 locus numbers in the “Description” column.

| ***Strains*** |  |  |  | | |  |
| --- | --- | --- | --- | --- | --- | --- |
| **Strain name** | **Organism** | **Genotype** | **Description** | | | **Source** |
| FC19 | *C. crescentus* CB15 | CB15 | Wild-type | | | ATCC 19089 |
| FC1365 | *C. crescentus* CB15 | ∆*hfiA* | In-frame deletion of *CC_0817* | | | Ref. 10 |
| FC1974 | *C. crescentus* CB15 | ∆*hfsJ* | In-frame deletion of *CC_0095* | | | Ref. 10 |
| FC3105 | *C. crescentus* CB15 | ∆*pleD* | In-frame deletion of *CC_2462* | | | This work |
| FC3054 | *C. crescentus* CB15 | ∆*CCNA_00497* | In-frame deletion of *CC_0465* | | | This work |
| FC3055 | *C. crescentus* CB15 | ∆*CCNA_02386* | In-frame deletion of *CC_2301* | | | This work |
| FC3056 | *C. crescentus* CB15 | ∆*rfbB* | In-frame deletion of *CC_3629* | | | This work |
| FC3057 | *C. crescentus* CB15 | ∆*wbqP* | In-frame deletion of *CC_1486* | | | This work |
| FC3058 | *C. crescentus* CB15 | ∆*hfsJ* ∆*wbqP* | In-frame deletion of *CC_1486* in FC1974 background | | | This work |
| FC1266 | *C. crescentus* CB15 | ∆*flgH* | In-frame deletion of *CC_2066* | | | This work |
| FC3013 | *C. crescentus* CB15 | ∆*cpaH* | In-frame deletion of *CC_2940* | | | This work |
| FC1265 | *C. crescentus* CB15 | ∆*pilA* | In-frame deletion of *CC_2948* | | | This work |
| FC3019 | *C. crescentus* CB15 | ∆*CCNA_01242* | In-frame deletion of *CC_1184* | | | This work |
| FC3021 | *C. crescentus* CB15 | ∆*hfsL* | In-frame deletion of *CC_2277* | | | This work |
| FC3020 | *C. crescentus* CB15 | ∆*hfaE* | In-frame deletion of *CC_2639* | | | This work |
| FC3015 | *C. crescentus* CB15 | ∆*hfsJ* ∆*flgH* | In-frame deletion of *CC_2066* in FC1974 background | | | This work |
| FC3016 | *C. crescentus* CB15 | ∆*hfsJ* ∆*cpaH* | In-frame deletion of *CC_2940* in FC1974 background | | | This work |
| FC3107 | *C. crescentus* CB15 | ∆*flgH* ∆*cpaH* | In-frame deletion of *CC_2940* in FC1266 | | | This work |
| FC3085 | *C. crescentus* CB15 | ∆*flgH* ∆*hfiA* | In-frame deletion of *CC_0817* in FC1266 background | | | This work |
| FC3083 | *C. crescentus* CB15 | ∆*cpaH* ∆*hfiA* | In-frame deletion of *CC_0817* in FC3013 background | | | This work |
| FC3084 | *C. crescentus* CB15 | ∆*pilA* ∆*hfiA* | In-frame deletion of *CC_0817* in FC1265 background | | | This work |
| FC3104 | *C. crescentus* CB15 | ∆*flgH* ∆*pleD* | In-frame deletion of *CC_2462* in FC1266 background | | | This work |
| FC3103 | *C. crescentus* CB15 | ∆*cpaH* ∆*pleD* | In-frame deletion of *CC_2462* in FC3013 background | | | This work |
| FC3108 | *C. crescentus* CB15 | ∆*pilA* ∆*pleD* | In-frame deletion of *CC_2462* in FC1265 background | | | This work |
| FC3017 | *C. crescentus* CB15 | ∆*flgH* ∆*pilA* | In-frame deletion of *CC_2948* in FC1266 background | | | This work |
| FC3018 | *C. crescentus* CB15 | ∆*cpaH* ∆*pilA* | In-frame deletion of *CC_2948* in FC3013 background | | | This work |
| FC3097 | *C. crescentus* CB15 | ∆*CCNA_00497 xyl::P_xyl_-empty* | pXGFPC-2 integrated at xylose locus of FC3054 | | | This work |
| FC3098 | *C. crescentus* CB15 | ∆*CCNA_00497 xyl::P_xyl_-CCNA_00497* | pFC3080 integrated at xylose locus of FC3054 | | | This work |
| FC3101 | *C. crescentus* CB15 | ∆*CCNA_02386 xyl::P_xyl_-empty* | pXGFPC-2 integrated at xylose locus of FC3055 | | | This work |
| FC3102 | *C. crescentus* CB15 | ∆*CCNA_02386 xyl::P_xyl_-CCNA_02386* | pFC3082 integrated at xylose locus of FC3055 | | | This work |
| FC3095 | *C. crescentus* CB15 | ∆*rfbB xyl::P_xyl_-empty* | pXGFPC-2 integrated at xylose locus of FC3056 | | | This work |
| FC3096 | *C. crescentus* CB15 | ∆*rfbB xyl::P_xyl_-rfbB* | pFC3079 integrated at xylose locus of FC3056 | | | This work |
| FC3099 | *C. crescentus* CB15 | ∆*wbqP xyl::P_xyl_-empty* | pXGFPC-2 integrated at xylose locus of FC3057 | | | This work |
| FC3100 | *C. crescentus* CB15 | ∆*wbqP* *xyl::P_xyl_-wbqP* | pFC3081 integrated at xylose locus of FC3057 | | | This work |
| FC3075 | *C. crescentus* CB15 | ∆*flgH* *xyl::P_flgE_-empty* | pFC3094 integrated at xylose locus of FC1266 | | | This work |
| FC3074 | *C. crescentus* CB15 | ∆*flgH xyl::P_flgE_-flgH* | pFC3093 integrated at xylose locus of FC1266 | | | This work |
| FC3070 | *C. crescentus* CB15 | ∆*cpaH xyl::P_xyl_-empty* | pXGFPC-2 integrated at xylose locus of FC3013 | | | This work |
| FC3071 | *C. crescentus* CB15 | ∆*cpaH xyl::P_xyl_-cpaH* | pFC3090 integrated at xylose locus of FC3013 | | | This work |
| FC3073 | *C. crescentus* CB15 | ∆*pilA xyl::P_pilA_-empty* | pFC3092 integrated at xylose locus of FC1265 | | | This work |
| FC3072 | *C. crescentus* CB15 | ∆*pilA* *xyl::P_pilA_-pilA* | pFC3091 integrated at xylose locus of FC1265 | | | This work |
| FC3126 | *C. crescentus* CB15 | ∆*CCNA_01242 xyl::P_CCNA_01242_-empty* | pFC3124 integrated at xylose locus of FC3019 | | | This work |
| FC3127 | *C. crescentus* CB15 | ∆*CCNA_01242 xyl::P_CCNA_01242_-CCNA_01242* | pFC3125 integrated at xylose locus of FC3019 | | | This work |
| FC3088 | *C. crescentus* CB15 | ∆*hfsL xyl::P_xyl_-empty* | pXGFPC-2 integrated at xylose locus of FC3021 | | | This work |
| FC3089 | *C. crescentus* CB15 | ∆*hfsL* *xyl::P_xyl_-hfsL* | pFC3078 integrated at xylose locus of FC3021 | | | This work |
| FC3086 | *C. crescentus* CB15 | ∆*hfaE xyl::P_xyl_-empty* | pXGFPC-2 integrated at xylose locus of FC3020 | | | This work |
| FC3087 | *C. crescentus* CB15 | ∆*hfaE xyl::P_xyl_-hfaE* | pFC3077 integrated at xylose locus of FC3020 | | | This work |
| APA_752 | *E. coli* WM3064 | Tn-HiMar (Km^R^) | Bacroded transposon pool for created BarSeq libraries | | | Ref. 29 |
|  |  |  |  | | |  |
| ***Plasmids*** |  |  |  | | |  |
| **Plasmid name** | **Description** | | | **Antibiotic** | **Reference** | |
| pNPTS138 | Suicide plasmid for making unmarked deletions in *C. crescentus*; carries sacB for counter-selection | | | Km | M. R Alley  unpublished | |
| pFC3059 | To delete *CCNA_01242*; contains fusion of *CC_1184* flanking regions with first and last 12 nucleotides of *CC_1184* ORF included | | | Km | This work | |
| pFC3060 | To delete *hfaE*; contains fusion of *CC_2639* flanking regions with first and last 12 nucleotides of *CC_2639* ORF included | | | Km | This work | |
| pFC3061 | To delete *hfsL*; contains fusion of *CC_2277* flanking regions with first 12 and last 96 nucleotides of *CC_2277* ORF included | | | Km | This work | |
| pFC3063 | To delete *CCNA_00497*; contains fusion of *CC_0465* flanking regions with first and last 12 nucleotides of *CC_0465* ORF included | | | Km | This work | |
| pFC3062 | To delete *CCNA_02386*; contains fusion of *CC_2301* flanking regions with first and last 12 nucleotides of *CC_2301* ORF included | | | Km | This work | |
| pFC3068 | To delete *rfbB*; contains fusion of *CC_3629* flanking regions with first and last 12 nucleotides of *CC_3629* ORF included | | | Km | This work | |
| pFC3069 | To delete *wbqP*; contains fusion of *CC_1486* flanking regions with first and last 12 nucleotides of *CC_1486* ORF included | | | Km | This work | |
| pFC1267 | To delete *pilA*; contains fusion of *CC_2948* flanking regions with first and last 12 nucleotides of *CC_2948* ORF included | | | Km | This work | |
| pFC1268 | To delete *flgH*; contains fusion of *CC_2066* flanking regions with first and last 12 nucleotides of *CC_2066* ORF included | | | Km | This work | |
| pFC3065 | To delete *cpaH*; contains fusion of *CC_2940* flanking regions with first and last 12 nucleotides of *CC_2940* ORF included | | | Km | This work | |
| pFC3067 | To delete *pleD*; contains fusion of *CC_2462* flanking regions with first and last 12 nucleotides of*CC_2462* ORF included | | | Km | This work | |
| pXGFPC-2  (pMT585) | Contains multiple cloning site downstream of Pxyl; integrates upstream of *xylX*; used for complementations | | | Km | Ref. 50 | |
| pFC3077 | pXGFPC-2 containing *hfaE* under the control of *P_xyl_* for integration at *xylX* locus | | | Km | This work | |
| pFC3078 | pXGFPC-2 containing *hfsL* under the control of *P_xyl_* for integration at *xylX* locus | | | Km | This work | |
| pFC3080 | pXGFPC-2 containing *CCNA_00497* under the control of *P_xyl_* for integration at *xylX* locus | | | Km | This work | |
| pFC3082 | pXGFPC-2 containing *CCNA_02386* under the control of *P_xyl_* for integration at *xylX* locus | | | Km | This work | |
| pFC3079 | pXGFPC-2 containing *rfbB* under the control of *P_xyl_* for integration at *xylX* locus | | | Km | This work | |
| pFC3081 | pXGFPC-2 containing *wbqP* under the control of *P_xyl_* for integration at *xylX* locus | | | Km | This work | |
| pFC3091 | pXGFPC-2 containing *pilA* under the control of *P_pilA_* for integration at *xylX* locus; 316bp upstream of *CC_2948* fused to the *CC_2948* ORF was inserted in reverse oreintation into pXGFPC-2 | | | Km | This work | |
| pFC3092 | pXGFPC-2 containing *P_pilA_* without insert for integration at *xylX* locus; 316bp upstream of *CC_2948* was inserted in reverse orientation into pXGFPC-2 | | | Km | This work | |
| pFC3093 | pXGFPC-2 containing *flgH* under the control of *P_flgF_* for integration at *xylX* locus; 226bp upstream of *CC_2063* fused to the *CC_2066* ORF was inserted in reverse oreintation into pXGFPC-2 | | | Km | This work | |
| pFC3094 | pXGFPC-2 containing *P_flgE_* without insert for integration at *xylX* locus; 216bp upstream of *CC_2063* was inserted in reverse orientation into pXGFPC-2 | | | Km | This work | |
| pFC3090 | pXGFPC-2 containing *cpaH* under the control of *P_xyl_* for integration at *xylX* locus | | | Km | This work | |
| pFC3124 | pXGFPC-2 containing *P_CCNA_01242_* without insert for integration at *xylX* locus; 99bp upstream of the *CC_1184* ORF was inserted in reverse orientation into pXGFPC-2 | | | Km | This work | |
| pFC3124 | pXGFPC-2 containing *CCNA_01242* under the control of *P_CCNA_01242_* for integration at *xylX* locus; 99bp upstream of *CC_1184* fused to the *CC_1184* ORF was inserted in reverse oreintation into pXGFPC-2 | | | Km | This work | |
| pFC1948 | pRKlac290 containing the *hfiA* promoter fused to *lacZ* | | | Tet | Ref. 10 | |
